# Checkpoint molecules on infiltrating immune cells in colorectal tumor microenvironment

**DOI:** 10.3389/fmed.2022.955599

**Published:** 2022-08-22

**Authors:** Iman M. Talaat, Noha M. Elemam, Shroque Zaher, Maha Saber-Ayad

**Affiliations:** ^1^Department of Clinical Sciences, College of Medicine, University of Sharjah, Sharjah, United Arab Emirates; ^2^Sharjah Institute for Medical Research, University of Sharjah, Sharjah, United Arab Emirates; ^3^Pathology Department, Faculty of Medicine, Alexandria University, Alexandria, Egypt; ^4^College of Medicine, Mohammed Bin Rashid University of Medicine and Health Sciences, Dubai, United Arab Emirates; ^5^Department of Pharmacology, Faculty of Medicine, Cairo University, Cairo, Egypt

**Keywords:** tumor microenvironment, macrophages, neutrophils, lymphocytes, colorectal cancer, immune checkpoint

## Abstract

Colorectal cancer (CRC) is one of the most prevalent cancer types worldwide, with a high mortality rate due to metastasis. The tumor microenvironment (TME) contains multiple interactions between the tumor and the host, thus determining CRC initiation and progression. Various immune cells exist within the TME, such as tumor-infiltrating lymphocytes (TILs), tumor-associated macrophages (TAMs), and tumor-associated neutrophils (TANs). The immunotherapy approach provides novel opportunities to treat solid tumors, especially toward immune checkpoints. Despite the advances in the immunotherapy of CRC, there are still obstacles to successful treatment. In this review, we highlighted the role of these immune cells in CRC, with a particular emphasis on immune checkpoint molecules involved in CRC pathogenesis.

## Introduction

Colorectal cancer (CRC) is the third most commonly diagnosed cancer in 2020, affecting 10% of the global population (1). The increasing mortality rate in patients with advanced CRC is of concern and reflects the limited range of treatment options. This could be attributed to the diagnosis of CRC at a late stage when the tumor has already metastasized. Furthermore, in most CRC patients, surgical resections are not the ultimate cure as there is a high possibility of recurrence of the disease in a more aggressive form; thus, using additional therapeutic modalities is mandatory (2). CRC is not a single disease and every patient has a unique illness due to distinctive genetic/epigenetic causes (3). The molecular classification of CRC is changing over time. Global genomic status [microsatellite instability (MSI) status and chromosomal instability (CIN) status] and epigenomic status [CpG island methylator phenotype (CIMP) status] contribute significantly to the clinical, pathological and biological properties of CRC. CIN tumors are mostly microsatellite stable (MSS) and have been associated with an aggressive clinical picture (4–6). Such tumors usually have large genomic abnormalities that lead to higher average DNA copy number compared with MSI tumors (7). MSI is typically diagnosed by the variable lengths of DNA microsatellites (mononucleotide and dinucleotide repeats) (8), which are caused by epigenetic silencing (9, 10) or mutation of DNA mismatch repair (MMR) genes, leading to accumulated mutations at 10–100 times the normal rate promoting cancer progression (8). CRC tumourigenesis has been reported to be triggered by gene mutations associated with multiple signaling pathways such as KRAS, BRAF, and PIK3CA (11). Several studies have confirmed that association between BRAF and KRAS mutations, in addition to BRAF mutations being more linked to MSI status (3, 12–14).

The tumor microenvironment (TME) is a dynamic and ever-changing phenomenon that has a pivotal role in determining CRC initiation and progression. The TME is a unique environment that develops during tumor progression due to its interactions with the host. It comprises several components, such as immune cells, stromal cells, myofibroblasts, vessels, and extracellular matrix (ECM), which differ according to tumor type (15). The tumor growth occurs in a multi-step process, where the neoplastic cells recruit stromal and immune cells to establish the TME. Then, within the tumor site, the deranged production of inflammatory cytokines and growth factors by cellular components in the TME leads to further recruitment of various immune cells (16). Finally, angiogenesis and ECM degradation occur during the tumor growth, eventually leading to invasion and metastasis. Several multiplexed technologies, such as single-cell RNA sequencing and mass cytometry, explore the functional diversities of tumor-infiltrating immune cells and the recent progress in the cancer immunotherapy (17). Furthermore, multiplex immunohistochemistry/immunofluorescence (mIHC/IF) provides throughput staining and standardized quantitative analysis that could be a proficient approach to detect specific proteins or molecular aberrations as well as explore the immune evasion (18). Thus, it could have a great potential to discover novel prognostic and predictive biomarkers in cancer immunotherapy and contribute in translational research and clinical practice (19). During multiplex IHC, more than three markers can be analyzed simultaneously in a single cut of formalin fixed parrafin embedded tissue (FFPE) with good cell discrimination and spatial information due to recent developments in multiple immunolabeling and multispectral imaging (20–23). A valuable method for assessing the expression of numerous markers simultaneously in a single tissue section was a multiplex IHC with tyramide signal amplification (TSA) (20–24). This is a more sensitive method than standard chromogenic IHC and may be able to identify proteins that are expressed at lower quantities (20, 25). In this review, we aim to discuss the various cellular immune components, focusing on the impact of immune checkpoint molecules on the CRC TME.

## Immune checkpoint molecules

The therapeutic use of antibodies that disrupt immune checkpoints was a critical turning point in the cancer immunotherapy (26). Blocking inhibitory coreceptors and pathways, which constrain immune cell activities in normal physiologic contexts, might “loosen the brakes” on immunological response, thus eliminating tumors. Immune cell activities are known to be exploited in malignancies (27). In addition, multiple immune checkpoint molecules have been identified in CRC pathogenesis and on various cell types, including lymphocytes, macrophages and neutrophils (28).

The co-inhibitory receptor programmed death-1 (PD-1), also known as CD279, is expressed inducibly on CD4^+^ T cells, CD8^+^ T cells, B cells, natural killer T cells, and macrophages (29). PD-L1 (B7-H1) and PD-L2 (B7-DC) are two known PD-1 ligands. PD-L1 is constitutively expressed on various immune and non-immune cells. However, PD-L2 expression can be induced in response to microenvironmental stimuli (30). The upregulation of PD-1 on tumor-infiltrating lymphocytes (TILs) and the increased expression of its ligands on tumor cells have been linked to tumor immune evasion, resulting in the suppression of tumor-specific CD8^+^ T cells. This receptor upregulation has also been linked to T cell exhaustion in malignant tumors, defined as a reduction in the proliferation and cytokine production (31). Thus, blocking PD-1 and PD-L1 using monoclonal antibodies (mAbs) might be effective in stage IV solid tumors by overcoming this immune suppression (32, 33).

A well-known immune checkpoint molecule is cytotoxic T lymphocyte antigen-4 (CTLA-4), expressed on T lymphocytes’ surfaces. CTLA-4 binds to B7-1 (CD80) and B7-2 (CD86) costimulatory receptors present on antigen-presenting cells (APCs), leading to inhibition of T cell activity by competitive blocking of CD28 (29). Therefore, CTLA-4 has been a hot target for mAbs cancer immunotherapy such as Ipilimumab (28). A remarkable target for immune checkpoint blockade (ICB) is lymphocyte activation gene-3 (LAG-3), a surface molecule of the immunoglobulin superfamily. LAG-3 interacts with MHC class II markers, thus leading to negative regulation of T cells, natural killer (NK) cells, B cells, and plasmacytoid dendritic cells (DCs) (34, 35). T cell immunoglobulin and mucin-containing protein-3 (TIM-3) is another immune checkpoint marker expressed on T helper 1 (Th1) and CD8^+^ cytotoxic T cells (CTLs). TIM-3 plays a critical role in inhibiting Th1 responses by causing cell death and is also known as hepatitis A virus cellular receptor 2 (HAVCR2) (36). Hence, blocking TIM-3 boosted the anti-tumor activity, with a greater efficiency upon combinatorial effect with PD-1 blockade (36). On the other hand, blockage of the inducible T-cell co-stimulator (ICOS), belonging to the B7-CD28 immunoglobulin superfamily, gained promising results in the treatment of different malignancies. Its expression is linked to a better prognosis in CRC patients, as the percentage of ICOS^+^ CD4^+^ cells operating as Th1 cells in either primary tumor tissue or peripheral blood could be a clinical predictive marker for a favorable prognosis (37).

CD40, a member of the tumor necrosis factor (TNF) family, was characterized on immune cells such as DCs, B cells and macrophages, as well as non-immune cells. The ligand of CD40 (CD40L) is expressed by activated B and T cells as well as platelets (38). CD40/CD40L interactions regulate T cell activity, cytokine production and antigen presentation (38, 39). In some cases, this interaction could inhibit tumor growth (40). On the other hand, tumors could utilize the CD40/CD40L to manipulate both T-cell and antigen-presenting compartments, thus contributing to the establishment of the immunosuppressive TME (38, 41). For instance, this immunosuppression could be achieved by inducing their proliferative capacity, growth, and survival (42).

Sialic acid-binding immunoglobulin-type lectins (Siglecs) are expressed on most white blood cells of the immune system, as well as TILs, DCs, and macrophages. Hypersialylation of neoplastic cells was identified as a hallmark of poor clinical outcomes and contributes to tumor escape from immune surveillance (43). Therefore, they are considered potential immune checkpoint targets for anticancer therapy (44, 45). Another promising target for cancer immunotherapy is the T cell immunoreceptor with immunoglobulin and ITIM domain (TIGIT). Its expression was known to be upregulated by various immune cells such as activated T cells, regulatory T (Treg) cells and NK cells. In addition, it can bind to two known ligands, CD155 and CD112, expressed by tumor and antigen-presenting cells in the TME (46).

## Therapies targeting immune checkpoint molecules in colorectal cancer

Several immunotherapeutic strategies are under clinical trials, especially in metastatic CRC; however, the results in MSS-CRC are generally modest. The ongoing studies investigate the outcome and potential biomarkers of metastatic CRC using various immunotherapy-based modalities, including immune checkpoint blockers (ICB) such as PD-1 blockers (e.g., nivolumab, pembrolizumab, atezolizumab, avelumab, durvalumab) and CTLA-4 blockers (e.g., ipilimumab, tremelimumab). This is besides the use of other approaches such as cancer vaccines (autologous, peptide, viral vector, and dendritic cell-based) that aim to stimulate an immune response against tumor cells, as well as adoptive cell transfer using chimeric antigen receptor T-cell therapy to kill the tumor cells directly, and oncolytic virus therapy (e.g., herpes simplex virus and NV 1020) where the viruses selectively replicate in cancer cells to destroy them with no harm to normal cells. Also, among immunotherapies under clinical trials are indoleamine 2,3-dioxygenase 1 (IDO-1) inhibitors, OX40 antagonists (e.g., epacadostat, indoximod) that enhance the immune response, and biphasic antibody targeting carcinoembryonic antigen (e.g., RO6958688) on T cells (47, 48).

Multiple clinical trials in this research area are at different phases, and some of which have been completed and the results are expected to be published soon. To mention a few examples, a phase II clinical trial investigated a combination of pembrolizumab and azacytidine in metastatic CRC refractory to chemotherapy. The findings demonstrated the safety and tolerability of this regimen, however, the clinical effect was modest in the investigated cohort, likely due to DNA methylation and immunomodulation of the tumor as an effect of azacitidine therapy (NCT02260440) (49). Another remarkable study was IMblaze370, which did not meet its primary endpoint of improved overall survival with atezolizumab plus cobimetinib or monotherapy using atezolizumab vs. regorafenib in previously treated metastatic CRC (NCT02788279). The study findings highlighted the challenge of using immunotherapy in tumors with low baseline levels of immune inflammation, such as that observed in the MSS metastatic CRC (50). Results from ongoing comparative clinical trials, such as Morpheus-CRC, are likely to thoroughly evaluate the role of immunotherapy in CRC. Morpheus-CRC is an ongoing study to evaluate the efficacy and safety of multiple immunotherapy combinations in metastatic CRC (NCT03555149) (48).

There are several challenging factors in using immunotherapeutic agents in CRC. In contrast to melanoma, which represents a successful example of immunotherapy, patients with metastatic CRC responded modestly to immunotherapy treatment, with many trials with high failure rates. Several mechanisms may explain the discrepancy in immunotherapy outcomes in different types of cancer. The tumor mutational burden (TMB) has been early identified as a potential predictor for effective response to immunotherapy. For example, MSI in CRC, where there is deficient DNA repair, gives rise to high TMB. In addition, appropriate immune response in the intestine could be preserved by ameliorating the host immune system that must tolerate commensal bacteria while maintaining the ability to face infections, otherwise, severe chronic inflammatory reactions might occur (51). Another important aspect of the poor outcome of CRC to immunotherapy is the fact that most tumors are associated with activated WNT/β-catenin signaling which can promote dendritic cell and T-cell exhaustion (52). This is similar to metastatic melanoma, where the activation of the WNT/β-catenin signaling pathway resulted in T-cell exclusion and resistance to anti-PD-L1/anti-CTLA-4 monoclonal antibody immunotherapy (53). Similarly, in a mouse model of hepatocellular carcinoma, the β-catenin pathway enhanced immune escape and suppressed the recruitment of DCs, and consequently led to impaired T-cell activity (54). Apart from the MSI status of the tumor, at the moment, no predictive biomarkers of immunotherapy response in CRC are available.

## Immune components of the colon cancer microenvironment

**FIGURE 1:**
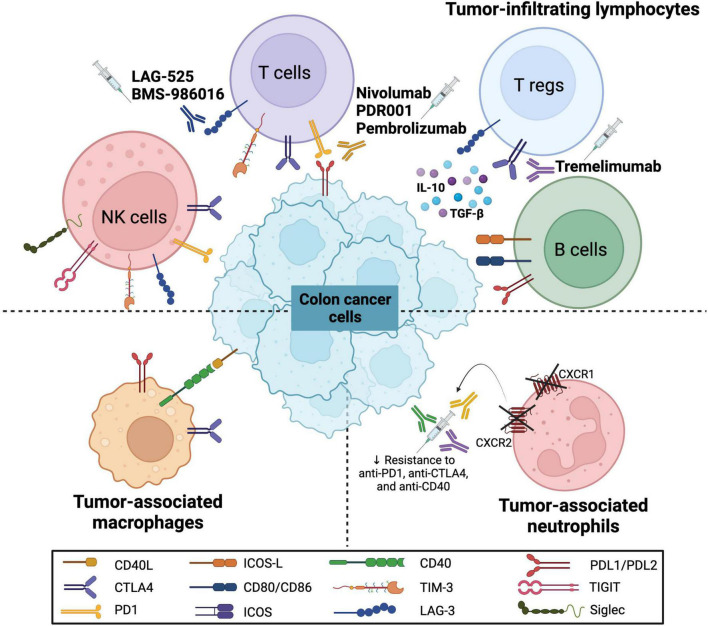
Immune checkpoint molecules on various immune cells in colorectal cancer. The schematic representation shows the expression of various immune checkpoint molecules on immune cells that interact with colon cancer cells. These immune cells include tumor-infiltrating lymphocytes (TILs) such as natural killer (NK) cells, T cells, regulatory T cells (Tregs) and B cells, as well as tumor-associated macrophages (TAMs) and tumor-associated neutrophils (TANs). The molecules include programmed cell death (PD1) and its ligand (PDL1/PDL2), CD40 and its ligand (CD40L), CD80/CD86, cytotoxic T-lymphocyte associated protein 4 (CTLA4), T-cell immunoglobulin and mucin domain 3 (TIM-3), lymphocyte-activation gene 3 (LAG-3), identification of the inducible T cell co-stimulator (ICOS) and its ligand (ICOS-L), T cell immunoreceptor with Ig and ITIM domains (TIGIT) and sialic acid-binding immunoglobulin-type lectins (Siglec). Furthermore, several monoclonal antibodies have been introduced to target these molecules (LAG-3, PD/PD-L axis, and CTLA4) as potential CRC immunotherapeutic agents.

The cellular landscape of the TME includes various immune cells, namely, TILs such as T, B, and NK cells, as well as tumor-associated macrophages (TAMs) and tumor-associated neutrophils (TANs). Various immune checkpoint molecules are expressed on these immune cells, thus modulating the colon cancer microenvironment and regulating the pathogenesis and response to therapy (Figure 1). The anti-tumor and pro-tumor roles of these immune cells on the TME have been previously discussed in CRC context [reviewed in (55)].

### Tumor-infiltrating lymphocytes

TILs mainly include CD8^+^ T cytotoxic and CD4^+^ T helper lymphocytes, in addition to B and NK cells. They are usually considered the host protecting element against tumor formation, as they induce the recruitment, maturation, and stimulation of immune cells that repress tumor growth (56).

#### T cells

In conventional terms, TILs represent the heterogeneous population of αβ T cells, both CD4^+^ and CD8^+^ subsets, present within the TME (57). CD8^+^ T cells (CTLs) recognize tumor-associated antigens (TAAs) along with proteins of HLA class I. These cells become differentiated into killer cells, release perforins, and express the apoptotic inducer FasL after expansion. Perforins disrupt the cell membrane, aiding the entry of granzymes inside the cells, causing cleavage of caspases’ precursors, thus directing the neoplastic cells toward apoptosis. Additionally, CD4^+^ T helper cells proved to have an essential role in the anti-tumor immunity by responding to antigens presented by antigen-presenting cells (APCs) such as macrophages (58).

Increased TILs is a favorable prognostic factor in many malignancies, including CRC (59). In addition, the quantification of lymphocyte infiltration has prognostic significance, suggesting that lymphocyte infiltration is not passive but may actively modulate tumor growth (60). This was supported by a large multicenter study spanning more than 10 years, which demonstrated levels of lymphocyte infiltration into primary tumors to be a strong independent predictor of relapse and overall survival (61). Using expression profiling of CRC, they further defined the relevance of specific immune signatures, demonstrating that Th1 type interferon-γ (IFN-γ) dominant immune profiles signified an improved prognosis. In contrast, Th17 type IL-17 dominant immune profiles signified a poor prognosis (61).

A recent study of most tumor-infiltrating immune cell subtypes revealed that CD8^+^ T cells had the most significant impact on patients’ survival (62). CD8^+^ CTLs mediate tumor rejection by recognizing TAAs and directly killing transformed cells. Effector CD8^+^ T cells in the TME generate IL-2, IL-12, and IFN-γ, which enhance the cytotoxic potential of CD8^+^ CTLs, leading to a targeted tumor cell killing (63, 64). On the other hand, CD4^+^ helper T cells present in the TME are involved in activating CTLs against tumor cells (65). Exhaustion of CTLs could be caused by long-term interaction between CTLs and antigens, leading to loss of their efficiency and function.

Similarly, tumor cells suppress the immune response by inducing the exhaustion of CTLs in the TME through the expression of inhibitory immune checkpoint receptors such as PD-1, CTLA-4, and LAG-3 (66, 67). In CRC pathogenesis, PD-1 was shown to be upregulated on CD8^+^ T cells in the TME, and its ligand was associated with cytokines and perforin impairment (30). Furthermore, a study by Hua et al. reported an inverse relationship between T cell density in the TME and the expression of PD-L1 on CRC cells (68). This was accompanied by an expansion of Treg cells, further linking the presence of PD-L1^+^ tumor cells and poor prognosis (68).

CTLA-4 was found to be expressed on TILs within the epithelial component of the tumor, the surrounding tumor stroma and the invasive front of the tumor. Further, CTLA-4 was identified on subsets of Treg cells, where high expression of CTLA-4 was revealed along with a significant increase of activated Tregs (CD45R Foxp3^+^ T cells) in the blood and tissues of CRC patients (69). Also, a highly suppressive subset of the CD4^+^ Foxp3^–^ T cell population was described in CRC patients to express multiple immune checkpoints (such as LAG-3, PD-1, and CTLA-4) and produce immunosuppressive cytokines such as IL-10 and transforming growth factor (TGF)-β (70). Therefore, CTLA-4 expression on Treg cells highlighted its potential role as a therapeutic target in CRC, such as in the case of Tremelimumab, which has been investigated in a phase II study for CRC patients with refractory metastatic adenocarcinoma who failed standard chemotherapy (70). Additionally, LAG-3 was reported to regulate the function of Treg cells, and its expression on CD4^+^ CD25^+^ cells was associated with potent inhibitory activity (71). Exhausted CD8^+^ T cells were observed to express LAG-3 along with other inhibitory receptors, such as PD-1, and thus inhibition of both PD-1 and LAG-3 could boost T cell activity (72). There are several clinical trials with LAG-3 inhibitors (LAG-525 and BMS-986016) with or without the combination of PD-1 inhibitors (Nivolumab and PDR001) in patients with advanced solid malignancies (28).

Xu et al. found considerably greater levels of circulating TIM-3^+^ PD-1^+^ CD8^+^ T cells in CRC patients’ peripheral blood samples than in healthy subjects’ blood (73). The expression of TIM-3 and PD-1 on CD8^+^ and CD4^+^ T cells was also revealed in peripheral blood collected after surgery. Furthermore, both TIM-3 and PD-1 expression appeared to be linked to decreased T cell activity (74). In comparison to adjacent colonic tissues, tumor tissue had a higher number of TIM-3^+^ PD-1^+^ CD8^+^ T cells. Together with the lack of quantifiable responses to PD-1 blockage in a large group of CRC patients, these findings point to TIM-3 as a more prominent inhibitory receptor in CRC patients, thus limiting T cell responses. Furthermore, inhibiting this route may help to restore damaged cell-mediated immunity following surgical resection. These findings support the development of TIM-3 inhibitors and show considerable promise in CRC patients as single or combined treatments (34).

Immunoregulatory cells such as Treg cells, mesenchymal derived stem cells (MDSCs), and M2 macrophages possess the ability to control and modulate T cell function by releasing cytokines such as IL-10 and TGF-β that can activate specific inhibitory immune checkpoints (75–77). Likewise, tumor cells and other cells in the TME can express these inhibitory ligands and activate their receptors, thus impairing T cells’ activity (78). This was reported to disrupt the proliferation of CTLs and reduce the immune response against CRC (79).

A known prognostic approach for immune checkpoint inhibitor therapy is MSI. Furthermore, MSI is linked with an MMR system that recognizes and repairs DNA damage. Several clinical trial data highlight that deficient MMR (dMMR) or MSI were able to predict treatment response across different solid tumor types, including CRC (80). In particular, MSI is known to be a good predictor of CRC prognosis, as it is closely associated with the abundance of tumor-infiltrating T cells. Several immunohistochemical studies have revealed high infiltration of intraepithelial activated CD8^+^ T cells within MSI colorectal tumors (81–83). Furthermore, Dolcetti et al. found that cytotoxic infiltrating structures were highly abundant in tumor epithelial cells of MSI-high (MSI-H) patients. The exact pathophysiology of TILs accumulation in MSI-H CRC has not been elucidated. However, an early proposal was that MSI-H tumors produce many abnormal proteins that trigger a host immune response. This was supported in a study by Smyrk et al. which reported an active immune microenvironment in MSI/dMMR tumors that are characterized by a more favorable prognosis compared to MSS/MMR-proficient (pMMR) tumors (8). In the MSI/dMMR subset of CRC, the high accumulated mutation creates many tumor-specific neoantigens, typically 10–50 times that of MSS/MMR-proficient subset (84), which might be the reason for the high level of TILs and active Th1/CTL immune microenvironment in MSI/MMR-tumors observed in many previous studies (8).

Moreover, granase B expression and other cytotoxic effects were more active in MSI-H tumors (85). Additionally, pMMR-microsatellite instability-low (MSI-L)/MSS have low tumor mutational burden, poor infiltration by TILs and often have a worse prognosis than dMMR-MSI-H as well as a poor response to immune checkpoint inhibitors (86). In the TME, the PD/PD-L1 pathway leads to the escape of tumor cells from the immune response via the inhibition of CTLs (87, 88). Additionally, the expression of PD-L1 on tumor cells is related to the exhaustion of T cells, therefore blocking this pathway has been demonstrated to be a successful approach for the treatment of different types of cancers, including non-small cell lung cancer, melanoma, breast, renal cell carcinoma, and CRC (87–92). In particular, higher expression of PD-1 and PD-L1 has been associated with a better prognosis in CRC patients. Furthermore, PD-1 expression in TILs has been found to be an independent prognostic factor for overall survival and disease-free survival of CRC patients, especially for MMR-proficient tumors (93). Therefore, the upregulation of the PD-1/PD-L1 axis in CRC is correlated with a favorable clinical outcome. Such a pattern could be a compensatory upregulatory mechanism in the TME in order to identify the tumor and trigger an immune response. Furthermore, an association between PD-L1 on tumor cells and a high TILs density could further support this hypothesis, similar to that observed in breast cancer (94, 95). Moreover, there is a remarkable high expression of checkpoint molecules such as PD-1, PD-L1, CTLA-4, and LAG-3 in MSI CRC in comparison to MSS CRC, which could contribute to the immunosuppressive microenvironment that aids MSI tumors evade immune destruction by the infiltrating immune cells. Therefore, this explains why the MSI subset of CRC could be a potentially good candidate for the checkpoint immunotherapy (9). ICB was described as more effective in MSI CRC in a phase 2 trial of Pembrolizumab, a fully human mAb targeting PD-1. In addition, another PD-1 mAb, Nivolumab, showed efficacy in CRC, where a patient showed complete response with no disease recurrence and demonstrated MSI (27, 96). Therefore, MMR status is a critical key for response to therapy, as shown by different clinical trials with anti-PD-1 and anti-PD-L1 therapy. Moreover, it was also demonstrated that CTLA-4 expression is increased in MSI tumors compared to MSS cancers (84).

#### B cells

Tumor-infiltrating B cells constitute a significant proportion of the immune infiltrates in CRC. Until recently, B cells have not been considered an important population of TILs, despite that they compose around 40% of TILs (97, 98). They are considered positive regulators of immunity, often collaborating with T cells to generate potent, unrelenting immune responses (98).

B cells can exert anti-tumor effects by activating antibody-dependent cell cytotoxicity (ADCC) and the complement cascade (99). In tumor tissues, B cells can be found in lymphoid aggregates, known as tertiary lymphoid structures (TLSs) or could be sparsely distributed in the TME. B cells present in the immature TLSs were reported to possess immune-regulatory functions by the secretion of anti-inflammatory cytokines and thus leading to the inhibition of anti-tumor immunity (100). Also, B cells can act as APCs besides their main function as antibody producers. Furthermore, B cells possess the unique capability of concentrating antigens through membrane immunoglobulin mediated uptake, which might also facilitate T cell activation above certain thresholds for TAAs (98, 101). Autoantibodies were shown to react primarily with autologous tumor targets or allogeneic tumors of the same tissue type, suggesting recognition of TAAs (102). Antibodies were believed to play a negligible role in the TME, so their relevance in tumor biology has been overlooked. However, studies revealed that B cell markers such as CD20 and CD138 correlated significantly with a lower CRC stage (103).

A study by Maletzki et al. observed that tumor-infiltrating B cells in primary CRC were of a mature immunophenotype, suggesting activation and antigen-induced maturation (104). This was supported by other studies where most tumor-infiltrating B cells reside in follicular aggregates in CRC. Likewise, peritumoral follicular aggregates of lymphocytes have been previously reported as a “Crohn’s-like reaction” and interpreted as an immune-mediated anti-tumor effect in CRC (105, 106). Similar to T cells, B cells express checkpoint ligands on their surface, such as PD-L1, CD80/CD86, and ICOS-L (107–109). Furthermore, a study by Helmink et al. observed significantly higher levels of B-cell-related gene expression, increased B cell receptor diversity, and clonal expansion in tumor samples from melanoma patients who responded to ICB treatment compared to other patients (110).

#### Natural killer cells

Being members of the innate immunity, NK cells can lyse tumor cells without prior sensitization or clonal expansion, unlike T cells. NK cells can be classified into two major groups, where the CD56*^bright^* CD16^–^ subset represents 10-15% of circulating NK cells and are more immunoregulatory by releasing cytokines such as IFN-γ. They mainly reside in the secondary lymphoid organs, such as lymph nodes and tonsils (111). In contrast, CD56*^dim^* CD16^+^ cells represent the significant population (90% of circulating NK cells) and predominantly mediate cytotoxicity (112, 113). NK cells play a fundamental role in cancer immunosurveillance through their anti-tumor activity (114). This has been supported by studies where the elimination of NK cells led to increased malignancy occurrence (115). NK cells perform their anti-tumor activity mainly when the expression of MHC class I molecules is downregulated. Moreover, upregulation of stress-induced molecules such as ligands of the activating receptor C type lectin receptor D (NKG2D) on cancerous cells makes them prone to NK-cell killing (116).

Most neoplastic cells and tumor-associated cells in the TME secrete factors that block the activation of NK cells, such as IL-6, IL-10, IDO, TGF-β, and prostaglandin E2 (PGE2), through downregulating NK cells activating receptors including NKG2D (117). Thus, NK cells, which infiltrate the tumor stroma, might proficiently lose their tumor-killing function due to these immunosuppressive mediators (118). For instance, IDO causes tryptophan depletion and kynurenine accumulation leading to immunosuppression of T and NK cell functions as well as the stimulation of Treg cells (119). Additionally, PGE2 suppresses IFN-γ production and responsiveness to IL-12 and IL-15 (120). Moreover, there is a reduction in the cytokine production of intra-tumoral NK cells (121). TGF-β affects the IL-15 signaling pathway, thus dampening NK cell proliferation and cytotoxicity (122). Furthermore, hypoxia and poor nutrient levels in the TME suppress NK cell activity (116). On another note, NK cell migration and penetration into the tumor growth site might be halted by ECM accumulation and increased interstitial fluid (123).

Furthermore, the recruitment of immunosuppressive cells such as MDSCs and the emergence of NK cell-resistant tumor variants result in primary tumor overgrowth. On the other hand, other tumor cells try to increase the expression of MHC class I molecules, such as human leukocyte antigen (HLA)-E, which engages the inhibitory receptor NKG2A on NK cells. This has been supported by studies where high expression of HLA-E and NKG2A led to a high inhibitory signal, potentially leading to poor outcomes and tumor growth (124–126).

NK cells have the potential to regulate the function of the adaptive immune system. For example, NK cells have been found to enhance T cell infiltration, thus triggering immune responses through their cytokine and chemokine secretion turning tumors immunologically “hot.” In contrast, the absence of these immune cells leaves the tumors immunologically “cold” (127). Consequently, CD8^+^ T cell recruitment in the TME and their interaction with NK cells elicit tumor regression. In addition, NK cells possess anti-metastatic activity by possible elimination of circulating tumor cells, “i.e., metastatic clones” (118, 127). However, tumors could escape NK cell activity through several mechanisms, including immune checkpoints expression by NK cells: PD-1, CTLA-4, LAG-3, and TIM-3. Upon binding to their receptors, NK cell activity is dampened (128), which can be surpassed by ICB, thus restoring NK and CD8^+^ T cell anti-tumor immunity. Nevertheless, many tumors still develop resistance to ICB therapy, representing a potential therapeutic target (129).

Another major obstacle in solid tumors is the homing of immune cells such as NK cells to tumor growth sites. This could be attributed to a dysregulation in the chemokine gradient in the TME, thus preventing NK cells from reaching the tumor growth sites (130). This has been reported in several studies where aberrant signaling pathways led to alterations in chemokines, including CCL27, CCL2, and CXCL11, hence impairing leukocyte migration (131–133). In CRC, loss of MHC class I expression is quite common, allowing NK cell recognition and killing of tumor cells (134, 135). However, like other types of cancer, a decreased number of NK cells in CRC patients was reported, which was associated with an increased frequency of CRC tumor recurrence (136, 137). This has been further supported where a negative correlation between peripheral NK cells and the CRC staging was reported, especially at early (I) and late (IV) stages of the disease (138). Phenotypically, CRC patients exhibited a reduction in the expression of the natural cytotoxicity receptors, NKp44 and NKp46 (139).

Furthermore, other activating receptors such as NKG2D, NKp30, NKp46, and DNAX accessory molecule-1 (DNAM-1) were reduced in the peripheral blood of patients with CRC (140–142). Upon tumor progression, the percentages of NKG2D^+^ NK cells were decreased, indicating a role in the metastasis of CRC (143). It has been shown that reduced expression of NKG2D on NK cells was correlated with high soluble serum levels of its ligand MHC-class I related molecule A (MICA) (144). The pathway of NKG2D and its ligands has been reported to be affected by TGF-β, which is highly expressed by colorectal cells (145). Hence, ligands of the activating receptor NKG2D were detected in the early stages of CRC, but as an immune evasion strategy, their expression decreased upon disease progression (146). Additionally, dysregulated NK cells displayed impaired function in CRC, including IFN-γ secretion and degranulation (140). Moreover, phenotypic alteration has been observed in the circulating CD56*^dim^* population of NK cells in CRC patients (139). Interestingly, a subpopulation of NK cells that is positive for CD16 and CD56 was studied and correlated negatively with the occurrence of CRC and the staging of CRC (147). The inhibitory receptor, NKG2A, has been reported to be an interesting target as a checkpoint molecule in cancer (148). Thus, blocking the inhibitory NKG2A receptor enhances tumor immunity by promoting both NK and CD8^+^ T cell effector functions. Monalizumab, a humanized anti-NKG2A antibody, was reported to induce NK cell activity against various tumor cells, especially in combination with PD axis blockade (149). This is under investigation in multiple clinical trials in solid tumors such as CRC (149).

Differentiated CRC cells were found to be more resistant to NK cells compared to cancer-initiating cells that were more susceptible to NK cell killing (150). It has been established by both *in vitro* and *in vivo* studies, where NK cells were shown to mediate the direct killing of human tumor cells in colon cancer (151–153). This has been implemented in clinical settings, where autologous NK cells were utilized in patients with advanced gastric or colorectal cancers combined with trastuzumab or cetuximab chemotherapy (154, 155). Colon adenocarcinomas exhibited low NK cell infiltration rates, thus causing the NK cell population to remain in the outer stroma and halting them from performing their anti-tumor activity (60, 134, 156, 157). Additionally, infiltration of NK cells was proposed to be a potential predictive marker of therapy. The homing and migration of NK cells are dependent on selectins, adhesion molecules and chemokines. Hence, future clinical trials should target the trafficking of NK cells into tumor sites rather than focusing on the simple administration of a single cytokine/chemokine as a therapeutic approach (157).

Another interesting aspect that is critical for immunotherapy for CRC is the expression of immune checkpoint molecules on NK cells (158). These include CTLA-4 and PD-1 receptors as well as TIGIT, CD96, LAG-3, and TIM-3. In CRC animal models and human patients, NK cell exhaustion was reported to be associated with the expression of TIGIT. Furthermore, the presence of NK cells was critical for the efficacy of TIGIT and PD-L1 checkpoint inhibitors, as they regulate the frequency of effector CD8^+^ T cells secreting IFN-γ and TNF-α (159). The combination of these checkpoint inhibitors showed a synergistic effect in their anti-tumor potential that was accompanied by prevention of NK cell exhaustion in both animal models and CRC patients (159, 160). In addition, PD-1 was found to be upregulated on tumor-infiltrating and peripheral NK cells in digestive cancers such as esophageal, gastric, biliary, and CRCs (161).

Other recently reported immune checkpoints are the Siglec family receptors, such as Siglec-7 and -9, CD47, and CD200. On another note, NK cells express Siglec-7 and Siglec-9 receptors, with a further upregulation on the cytotoxic CD56*^dim^* NK cell subset (162, 163). In addition, Siglec-9 was found to be upregulated on tumor-infiltrated CD8^+^ cytotoxic T cells in various solid tumors, including CRC (164, 165). An interesting fact about the Siglec immune checkpoint molecules is that they are expressed on various immune cells and are usually expressed on T cells that concomitantly express PD-1, further enhancing the co-inhibitory signal (165). Furthermore, they were known to play an inhibitory effect on NK cell function against tumor cells, particularly cytotoxicity.

On the other hand, blocking these immune checkpoint molecules such as Siglec-9 antibodies improved the anti-tumor cytotoxic potential of NK cells. This was due to the blockage of Siglec markers on tumor cells as well as the NKG2A receptor on NK cells (164). Also, sialidase treatment was found to enhance NK cell killing against various cell lines, including the colon cell lines. Therefore, anti-Siglec-7 and anti-Siglec-9 blocking antibodies could be developed to be used for cancer immunotherapy, along with other immune checkpoint inhibitors.

### Tumor-associated macrophages

TAMs are the dominant inflammatory constituent in the TME and are ample in all stages of carcinogenesis. Activated infiltrating TAMs secrete a plethora of proteolytic enzymes as well as growth and inflammatory mediators, known to modulate different molecular pathways involved in tumor progression and metastasis (166).

Macrophages can be classified into two well-defined subtypes: M1 macrophages “classically activated” and M2 macrophages “alternatively activated.” M1 macrophages have a pivotal role in eradicating different organisms and cancerous cells, as they have an inflammatory function by secreting pro-inflammatory cytokines like TNF-α, IL-6, and IL-1β. On the contrary, M2 macrophages release anti-inflammatory cytokines, such as TGF-β, IL-10, and IL-13, and have been implicated in tissue healing and tumor progression. M1 and M2 are distinguished with certain markers in the tumor samples, where M1 macrophages are characterized by the expression of HLA-DR, CD11c, CD86, inducible nitric oxide synthetase (iNOS), and phosphorylated signal transducer and activator of transcription 1 (pSTAT1), while M2 macrophages express CD163, CD204, and CD206 (167). In the TME, TAMs are mostly pro-tumorigenic/anti-inflammatory “M2 phenotype form.” Their significance in tumor evolution and progression is accentuated by the fact that they may comprise up to 80% of the tumor mass (168). The suppression of an immune response, activation of angiogenesis, and remodeling of ECM are important functional characteristics of TAMs. Furthermore, TAMs produce proteolytic enzymes such as matrix metalloproteinases (MMPs) and cathepsins that cause ECM breakdown, leading to the intravasation of tumor cells into the bloodstream, thus enhancing metastases (169). Additionally, TAMs release angiogenic factors, allowing tumor cells to spread beyond the primary tumor site and contributing to metastasis (170). They also provide a favorable environment for metastatic tumor cells by releasing inflammatory mediators like IL-1β. Furthermore, reactive oxygen species (ROS) produced by TAMs are implicated in malignant cell instability, a hallmark of cancer (168). On another note, TAMs could promote cancer cell proliferation by releasing growth factors such as epidermal growth factor (EGF) (170).

Recently, the effect of colon cancer ECM on macrophage polarization was investigated, where it was discovered that tumor ECM-educated macrophages could develop into M2 macrophages. The anti-inflammatory markers (IL-10, CCL18, and TGF-β) were upregulated, and the pro-inflammatory markers (TNF-α and IL-6) were downregulated by the macrophages that are differentiated within the tumor matrices. It was also found that MMP1, the MMP responsible for M2 polarization, was upregulated in tumor matrices. These results indicated that tumor-derived matrices caused an anti-inflammatory M2-like macrophage polarization significantly (171). Additionally, clinical staging and lymph node metastases were found to be associated with macrophage infiltration and vascular density in CRC (172). Moreover, blocking the colony-stimulating factor 1 receptor (CSF1R), required for TAMs’ recruitment, differentiation, and survival, is one of the most effective ways to target TAMs (173). Small molecule inhibitors or mAbs against CSF1R diminish the number and/or affect the behavior of TAMs in mice models of solid tumors such as CRC, breast cancer, and glioblastoma, thus impairing tumor formation and progression (174–176).

TAMs were reported to express molecular triggers of checkpoint proteins that regulate T-cell activation. Such proteins are the site of action of checkpoint-blockade immunotherapies (177). On another note, TAMs are key players in immunological resistance and their manipulation could improve the efficiency of immunotherapies, possibly through the NF-κB pathway. Such a pathway could be inhibited to increase the efficacy of immunotherapies by repolarizing M2 TAMs and to decrease the expression of PD-L1 on them (178). A recent study in CRC by Fiegle et al. showed that the combined blockade of CTLA-4 and PD-L1 increased the levels of the pro-inflammatory Th1/M1-related cytokines, increased NOS^+^ macrophages in the tumor tissue and reduced PD-L1^+^ macrophages (179). The role of TAMs as therapeutic targets was reviewed by Malfitano et al. (177). Also, CD40^+^ TAMs and plasma sCD40 in CRC tissues have been identified as favorable prognostic markers (180). Apoptotic susceptibility is dependent on the “quality” of the signal, as death occurs when the CD40 signal is delivered in membrane-bound form (mCD40L), whereas the soluble CD40 agonists are non-apoptotic (181). Blocking of CD40 using membrane-bound CD40L showed pro-apoptotic signal and pro-inflammatory cytokine production in CRC cells, thus suggesting CD40 as a promising therapeutic in CRC (182).

### Tumor-associated neutrophils

Neutrophils play an intricate and complex role in cancer (183). Many reports support the dual function of neutrophils, including anti-tumoral and pro-tumoral roles, and thus TANs are segregated into anti-tumor (N1) and pro-tumor (N2) phenotypes (184). However, these cells do not have specific cell surface markers to discriminate N1 and N2 neutrophils. Some studies indicate that N1 neutrophils have a higher expression of CD54, CD95, TNF-α, CXCL10, and low production of IL-8, while N2 neutrophils have high expression of CD182 and IL-8 production (185). In addition, neutrophils play a role in the immunosuppression of tumors (186), through the release of different mediators, including IL-4, TGF-β, immune checkpoint ligands, ROS, and reactive nitrogen intermediates (187). On the other hand, releasing nitric oxide by neutrophils could enhance cancer cell killing and suppress CRC growth and metastasis (188).

Under the effect of TGF-β present in the TME, neutrophils polarize into pro-tumor N2 neutrophils, which produce proangiogenic factors and exert immunosuppressive activity through the secretion of arginase-1 (Arg1) (184, 189, 190). TANs mediate direct suppression of Th1 and CTL in tumors (191). On the other hand, upon blockade of TGF-β or administration of type 1 IFN, neutrophils could polarize into anti-tumor N1 neutrophils, which activate CD8^+^ T cells, thus exerting anticancer cytotoxic activity, by reducing the expression of the proangiogenic factors (e.g., VEGF and MMP-9), and increasing the expression of T cell-attracting chemokines (e.g., CCL3, CXCL9, and CXCL10) (184, 189, 192).

Neutrophils are recruited to the tumor site through inflammatory molecules such as granulocyte-colony stimulating factor (G-CSF), tumor-derived cholesterol derivatives (oxysterols) (193) and anaphylatoxin C5a (complement component) (99, 194). In CRC, neutrophils play an anti-tumoral role through the secretion of IFN-β, IFN-γ and Granulocyte macrophage-colony stimulating factor (GM-CSF), and are known to express CD66b, CD11b, CD101, and CD177 (187). Neutrophils may promote tumor metastasis by accumulating in the metastatic niche. Tumor and stromal cells expressing G-CSF, CXCL1, and CXCL2 enhance neutrophil recruitment in the metastatic sites (195).

In solid tumors, neutrophils’ accumulation is a poor prognostic marker associated with tumor progression and metastases (196–198). However, in CRC, high infiltration of TANs was reported to be associated with a better response to 5-FU-based chemotherapy (199). In this regard, CRC represents an exception from other solid tumors in which a high number of TANs is associated with poor response to chemotherapy and radiotherapy (200). Different key players in tumor immunobiology among different cancers may explain the discrepancy of TANs function in CRC compared to other tumors (e.g., ovarian and gastric).

Noteworthy, neutrophils interact with TILs. Using an inducible colon tumor mouse model, Germann et al. reported that the most potent inhibitor of T-cell activity in the TME was the TANs. The suppression is exerted through matrix metalloproteinase-mediated activation of TGF-β (201). Interestingly, MMP-9 secreted by TANs, converts TGF-β precursor into an active form. Thus, inhibiting the MMP-9/TGF-β axis eliminates the immunosuppressive effect of neutrophils and suppresses their tumor-promoting functions (201). On the other hand, a recent study reported that the pre-operative and post-operative neutrophil to lymphocyte ratio was associated with histological markers of CRC progression. Also, there was a trend of association between post-operative neutrophil count and disease-free survival (202). Different factors affect neutrophil polarization and may, at least in part, explain the apparent paradoxical impact of TME neutrophil count.

The link between TANs infiltration and tumor angiogenesis determines to a great extent the response to ICBs. It has been reported that neutrophil infiltration in the TME is associated with significant resistance elements to ICBs and their adjuvant anti-angiogenic agents. More than 100 clinical trials investigate the combination of bevacizumab (Avastin; anti-VEGF-A antibody) with ICBs (203). In addition, inhibition of CXCL1 or CXCL5/CXCR2 signaling in tumors with low TILs causes a reduction in TANs infiltration, with an increase in the number of PD-1^+^ CD8^+^ T cells. Furthermore, this enhances the sensitization of cancer cells to the anti-CD40, anti-CTLA-4, and anti-PD-1 combination immunotherapy (204). Moreover, the use of CXCR2 inhibitors might overcome the resistance to anti-PD-1 immunotherapy in KRAS^*G*12D^-expressing CRC (205). Such findings, together with similar ones in other cancers, promoted the development of phase I and II clinical trials, using CXCR1 and CXCR2 inhibitors in combination with anti-PD-1 in patients with metastatic CRC with MSI-L and Ras-mutation (195). Furthermore, the “neutrophil extracellular trap” or “NET” is considered an important element of the TME that leads to resistance to ICB therapy (206, 207). Accordingly, DNase I, an inhibitor of NETs, was reported to significantly enhance the therapeutic effects of anti-PD-1 in an MC38-bearing mouse model of CRC (208).

## Conclusion

Blocking immune checkpoints has ushered in a new era of cancer treatment. Targeting immunological checkpoints in CRC TME is an intriguing novel cancer therapeutic approach via altering the immune cells’ function. Increasing evidence suggests that patients’ responses are linked to different pro-tumor and anti-tumor immune cells in the TME, such as TILs, TAMs, and TANs. Anti-PD-1, anti-PD-L1 and anti-CTLA-4 are well-known ICBs showing promising results in CRC patients. In addition, other intriguing immunological checkpoints that can suppress T or NK cell activity have emerged in recent years, such as TIM-3 and LAG-3. As a result, combining ICBs with other therapeutic modalities has shown encouraging results and could be a successful step forward in CRC treatment.

## Author contributions

All authors contributed to the conceptualization, drafting, and reviewing of the manuscript.

## References

[1] Sung H Ferlay J Siegel RL Laversanne M Soerjomataram I Jemal A Global Cancer Statistics 2020: GLOBOCAN estimates of incidence and mortality worldwide for 36 cancers in 185 countries. *CA Cancer J Clin.* (2021) 71:209–49. 10.3322/caac.21660 33538338

[2] Pretzsch E Bösch F Neumann J Ganschow P Bazhin A Guba M Mechanisms of metastasis in colorectal cancer and metastatic organotropism: hematogenous versus peritoneal spread. *J Oncol.* (2019) 2019:7407190. 10.1155/2019/7407190 31641356 PMC6770301

[3] Ogino S Goel A. Molecular classification and correlates in colorectal cancer. *J Mol Diagn.* (2008) 10:13–27. 10.2353/jmoldx.2008.070082 18165277 PMC2175539

[4] Walther A Johnstone E Swanton C Midgley R Tomlinson I Kerr D. Genetic prognostic and predictive markers in colorectal cancer. *Nat Rev Cancer.* (2009) 9:489–99. 10.1038/nrc2645 19536109

[5] Jass JR. Classification of colorectal cancer based on correlation of clinical, morphological and molecular features. *Histopathology.* (2007) 50:113–30. 10.1111/j.1365-2559.2006.02549.x 17204026

[6] Walther A Houlston R Tomlinson I. Association between chromosomal instability and prognosis in colorectal cancer: a meta-analysis. *Gut.* (2008) 57:941–50. 10.1136/gut.2007.135004 18364437

[7] Muzny DM Bainbridge MN Chang K Dinh HH Drummond JA Fowler G Comprehensive molecular characterization of human colon and rectal cancer. *Nature.* (2012) 487:330–7. 10.1038/nature11252 22810696 PMC3401966

[8] Smyrk TC Watson P Kaul K Lynch HT. Tumor-infiltrating lymphocytes are a marker for microsatellite instability in colorectal carcinoma. *Cancer.* (2001) 91:2417–22. 10.1002/1097-0142(20010615)91:123.0.CO;2-U 11413533

[9] Xiao Y Freeman GJ. The microsatellite instable subset of colorectal cancer is a particularly good candidate for checkpoint blockade immunotherapy. *Cancer Discov.* (2015) 5:16–8. 10.1158/2159-8290.Cd-14-1397 25583798 PMC4295637

[10] Ogino S Nosho K Kirkner GJ Kawasaki T Meyerhardt JA Loda M CpG island methylator phenotype, microsatellite instability, braf mutation and clinical outcome in colon cancer. *Gut.* (2009) 58:90–6. 10.1136/gut.2008.155473 18832519 PMC2679586

[11] Birgisson H Edlund K Wallin U Påhlman L Kultima HG Mayrhofer M Microsatellite instability and mutations in BRAF and KRAS are significant predictors of disseminated disease in colon cancer. *BMC Cancer.* (2015) 15:125. 10.1186/s12885-015-1144-x 25884297 PMC4364587

[12] Fariña-Sarasqueta A van Lijnschoten G Moerland E Creemers GJ Lemmens V Rutten HJT The BRAF V600e mutation is an independent prognostic factor for survival in stage II and Stage III colon cancer patients. *Ann Oncol.* (2010) 21:2396–402. 10.1093/annonc/mdq258 20501503

[13] Rajagopalan H Bardelli A Lengauer C Kinzler KW Vogelstein B Velculescu VE. Tumorigenesis: RAF/RAS oncogenes and mismatch-repair status. *Nature.* (2002) 418:934. 10.1038/418934a 12198537

[14] Gavin PG Colangelo LH Fumagalli D Tanaka N Remillard MY Yothers G Mutation profiling and microsatellite instability in stage II and III colon cancer: an assessment of their prognostic and oxaliplatin predictive value. *Clin Cancer Res.* (2012) 18:6531–41. 10.1158/1078-0432.Ccr-12-0605 23045248 PMC4273673

[15] Anderson NM Simon MC. The tumor microenvironment. *Curr Biol.* (2020) 30:R921–5. 10.1016/j.cub.2020.06.081 32810447 PMC8194051

[16] Wang M Zhao J Zhang L Wei F Lian Y Wu Y Role of tumor microenvironment in tumorigenesis. *J Cancer.* (2017) 8:761–73. 10.7150/jca.17648 28382138 PMC5381164

[17] Zhang Y Zhang Z. The history and advances in cancer immunotherapy: understanding the characteristics of tumor-infiltrating immune cells and their therapeutic implications. *Cell Mol Immunol.* (2020) 17:807–21. 10.1038/s41423-020-0488-6 32612154 PMC7395159

[18] Hofman P Badoual C Henderson F Berland L Hamila M Long-Mira E Multiplexed immunohistochemistry for molecular and immune profiling in lung cancer-just about ready for prime-time? *Cancers.* (2019) 11:283. 10.3390/cancers11030283 30818873 PMC6468415

[19] Tan WCC Nerurkar SN Cai HY Ng HHM Wu D Wee YTF Overview of multiplex immunohistochemistry/immunofluorescence techniques in the era of cancer immunotherapy. *Cancer Commun.* (2020) 40:135–53. 10.1002/cac2.12023 32301585 PMC7170662

[20] Stack EC Wang C Roman KA Hoyt CC. Multiplexed Immunohistochemistry, imaging, and quantitation: a review, with an assessment of tyramide signal amplification, multispectral imaging and multiplex analysis. *Methods.* (2014) 70:46–58. 10.1016/j.ymeth.2014.08.016 25242720

[21] Feng Z Puri S Moudgil T Wood W Hoyt CC Wang C Multispectral imaging of formalin-fixed tissue predicts ability to generate tumor-infiltrating lymphocytes from melanoma. *J Immunother Cancer.* (2015) 3:47. 10.1186/s40425-015-0091-z 26500776 PMC4617712

[22] FengZJensenSMMessenheimerDJFarhadMNeubergerMBifulcoCB Correction: multispectral imaging of T and B cells in murine spleen and tumor. *J Immunol.* (2017) 198:1759. 10.4049/jimmunol.1601990 28167652

[23] Stack EC Foukas PG Lee PP. Multiplexed tissue biomarker imaging. *J Immunother Cancer.* (2016) 4:1–3. 10.1186/s40425-016-0115-3 PMC4754920 26885371

[24] Huang W Hennrick K Drew SA. Colorful future of quantitative pathology: validation of vectra technology using chromogenic multiplexed immunohistochemistry and prostate tissue microarrays. *Hum Pathol.* (2013) 44:29–38. 10.1016/j.humpath.2012.05.009 22944297

[25] Gorris MAJ Halilovic A Rabold K van Duffelen A Wickramasinghe IN Verweij D Eight-color multiplex immunohistochemistry for simultaneous detection of multiple immune checkpoint molecules within the tumor microenvironment. *J Immunol.* (2018) 200:347. 10.4049/jimmunol.1701262 29141863

[26] Brahmer JR Pardoll DM. Immune checkpoint inhibitors: making immunotherapy a reality for the treatment of lung cancer. *Cancer Immunol Res.* (2013) 1:85–91. 10.1158/2326-6066.CIR-13-0078 24777499 PMC4856021

[27] Brahmer JR Drake CG Wollner I Powderly JD Picus J Sharfman WH Phase I study of single-agent anti–programmed death-1 (Mdx-1106) in refractory solid tumors: safety, clinical activity, pharmacodynamics, and immunologic correlates. *J Clin Oncol.* (2010) 28:3167–75. 10.1200/JCO.2009.26.7609 20516446 PMC4834717

[28] Jacobs J Smits E Lardon F Pauwels P Deschoolmeester V. Immune checkpoint modulation in colorectal cancer: what’s new and what to expect. *J Immunol Res.* (2015) 2015:158038. 10.1155/2015/158038 26605342 PMC4641952

[29] Greenwald RJ Freeman GJ Sharpe AH. The B7 family revisited. *Annu Rev Immunol.* (2004) 23:515–48. 10.1146/annurev.immunol.23.021704.115611 15771580

[30] Wu X Zhang H Xing Q Cui J Li J Li Y Pd-1+ Cd8+ T cells are exhausted in tumours and functional in draining lymph nodes of colorectal cancer patients. *Br J Cancer.* (2014) 111:1391–9. 10.1038/bjc.2014.416 25093496 PMC4183848

[31] Rozali EN Hato SV Robinson BW Lake RA Lesterhuis WJ. Programmed death ligand 2 in cancer-induced immune suppression. *Clin Dev Immunol.* (2012) 2012:656340. 10.1155/2012/656340 22611421 PMC3350956

[32] Kaufman HL Russell J Hamid O Bhatia S Terheyden P D’Angelo SP Avelumab in patients with chemotherapy-refractory metastatic merkel cell carcinoma: a multicentre, single-group, open-label, phase 2 trial. *Lancet Oncol.* (2016) 17:1374–85. 10.1016/s1470-2045(16)30364-3 27592805 PMC5587154

[33] D’Angelo SP Russell J Lebbé C Chmielowski B Gambichler T Grob JJ Efficacy and safety of first-line avelumab treatment in patients with stage IV metastatic merkel cell carcinoma: a preplanned interim analysis of a clinical trial. *JAMA Oncol.* (2018) 4:e180077. 10.1001/jamaoncol.2018.0077 29566106 PMC5885245

[34] Shin DS Ribas A. The evolution of checkpoint blockade as a cancer therapy: what’s here. What’s Next? *Curr Opin Immunol.* (2015) 33:23–35. 10.1016/j.coi.2015.01.006 25621841

[35] Goldberg MV Drake CG. Lag-3 in cancer immunotherapy. *Cancer Immunol Immunother.* (2010) 344:269–78. 10.1007/82_2010_114 PMC4696019 21086108

[36] Zhu C Anderson AC Schubart A Xiong H Imitola J Khoury SJ The tim-3 ligand galectin-9 negatively regulates t helper type 1 immunity. *Nat Immunol.* (2005) 6:1245–52. 10.1038/ni1271 16286920

[37] Zhang Y Luo Y Qin SL Mu YF Qi Y Yu MH The clinical impact of ICOS signal in colorectal cancer patients. *Oncoimmunology.* (2016) 5:e1141857. 10.1080/2162402x.2016.1141857 27467961 PMC4910717

[38] Elgueta R Benson MJ De Vries VC Wasiuk A Guo Y Noelle RJ. Molecular mechanism and function of Cd40/Cd40l engagement in the immune system. *Immunol Rev.* (2009) 229:152–72. 10.1111/j.1600-065X.2009.00782.x 19426221 PMC3826168

[39] Quezada SA Jarvinen LZ Lind EF Noelle RJ. Cd40/Cd154 interactions at the interface of tolerance and immunity. *Annu Rev Immunol.* (2004) 22:307–28. 10.1146/annurev.immunol.22.012703.104533 15032580

[40] Wu Y Wang L He X Xu H Zhou L Zhao F Expression of Cd40 and growth-inhibitory activity of Cd40 ligand in colon cancer ex vivo. *Cell Immunol.* (2008) 253:102–9. 10.1016/j.cellimm.2008.05.005 18603231

[41] Barth RJ Jr Fisher DA Wallace PK Channon JY Noelle RJ Gui J A randomized trial of ex vivo Cd40l activation of a dendritic cell vaccine in colorectal cancer patients: tumor-specific immune responses are associated with improved survival. *Clin Cancer Res.* (2010) 16:5548–56. 10.1158/1078-0432.CCR-10-2138 20884622 PMC2994719

[42] Honeychurch J Cheadle EJ Dovedi SJ Illidge TM. Immuno-regulatory antibodies for the treatment of cancer. *Exp Opin Biol Ther.* (2015) 15:787–801. 10.1517/14712598.2015.1036737 25882106

[43] Perdicchio M Ilarregui JM Verstege MI Cornelissen LA Schetters ST Engels S Sialic acid-modified antigens impose tolerance via inhibition of T-cell proliferation and de novo induction of regulatory T cells. *Proc Natl Acad Sci U.S.A.* (2016) 113:3329–34. 10.1073/pnas.1507706113 26941238 PMC4812702

[44] Duan S Paulson JC. Siglecs as immune cell checkpoints in disease. *Annu Rev Immunol.* (2020) 38:365–95. 10.1146/annurev-immunol-102419-035900 31986070

[45] Fraschilla I Pillai S. Viewing siglecs through the lens of tumor immunology. *Immunol Rev.* (2017) 276:178–91. 10.1111/imr.12526 28258691 PMC5860639

[46] Chauvin J-M Zarour HM. Tigit in cancer immunotherapy. *J Immunother Cancer.* (2020) 8:e000957. 10.1136/jitc-2020-000957 32900861 PMC7477968

[47] Tabernero J Melero I Ros W Argiles G Marabelle A Rodriguez-Ruiz ME Phase Ia and Ib studies of the novel carcinoembryonic antigen (CEA) T-cell bispecific (CEA CD3 TCB) antibody as a single agent and in combination with atezolizumab: preliminary efficacy and safety in patients with metastatic colorectal cancer (mCRC). *J Clin Oncol.* (2017) 35(15 Suppl.):3002. 10.1200/JCO.2017.35.15_suppl.3002 28644773

[48] Wrobel P Ahmed S. Current status of immunotherapy in metastatic colorectal cancer. *Int J Colorectal Dis.* (2019) 34:13–25. 10.1007/s00384-018-3202-8 30465238

[49] Kuang C Park Y Augustin RC Lin Y Hartman DJ Seigh L Pembrolizumab plus azacitidine in patients with chemotherapy refractory metastatic colorectal cancer: a single-arm phase 2 trial and correlative biomarker analysis. *Clin Epigenet.* (2022) 14:3. 10.1186/s13148-021-01226-y 34991708 PMC8740438

[50] Eng C Kim TW Bendell J Argilés G Tebbutt NC Di Bartolomeo M Atezolizumab with or without cobimetinib versus regorafenib in previously treated metastatic colorectal cancer (Imblaze370): a multicentre, open-label, phase 3, randomised, controlled trial. *Lancet Oncol.* (2019) 20:849–61. 10.1016/s1470-2045(19)30027-0 31003911

[51] Steimle A Frick JS. Molecular mechanisms of induction of tolerant and tolerogenic intestinal dendritic cells in mice. *J Immunol Res.* (2016) 2016:1958650. 10.1155/2016/1958650 26981546 PMC4766351

[52] Grasso CS Giannakis M Wells DK Hamada T Mu XJ Quist M Genetic mechanisms of immune evasion in colorectal cancer. *Cancer Discov.* (2018) 8:730–49. 10.1158/2159-8290.CD-17-1327 29510987 PMC5984687

[53] Spranger S Bao R Gajewski TF. Melanoma-intrinsic β-catenin signalling prevents anti-tumour immunity. *Nature.* (2015) 523:231–5. 10.1038/nature14404 25970248

[54] Ruiz de Galarreta M Bresnahan E Molina-Sánchez P Lindblad KE Maier B Sia D B-catenin activation promotes immune escape and resistance to anti-Pd-1 therapy in hepatocellular carcinoma. *Cancer Discov.* (2019) 9:1124–41. 10.1158/2159-8290.Cd-19-0074 31186238 PMC6677618

[55] Guo L Wang C Qiu X Pu X Chang P. Colorectal cancer immune infiltrates: significance in patient prognosis and immunotherapeutic efficacy. *Front Immunol.* (2020) 11:1052. 10.3389/fimmu.2020.01052 32547556 PMC7270196

[56] Angell H Galon J. From the immune contexture to the immunoscore: the role of prognostic and predictive immune markers in cancer. *Curr Opin Immunol.* (2013) 25:261–7. 10.1016/j.coi.2013.03.004 23579076

[57] Lavin Y Kobayashi S Leader A Amir ED Elefant N Bigenwald C Innate immune landscape in early lung adenocarcinoma by paired single-cell analyses. *Cell.* (2017) 169:750–65.e17. 10.1016/j.cell.2017.04.014 28475900 PMC5737939

[58] Kather JN Halama N. Harnessing the innate immune system and local immunological microenvironment to treat colorectal cancer. *Br J Cancer.* (2019) 120:871–82. 10.1038/s41416-019-0441-6 30936499 PMC6734657

[59] Nazemalhosseini-Mojarad E Mohammadpour S Torshizi Esafahani A Gharib E Larki P Moradi A Intratumoral infiltrating lymphocytes correlate with improved survival in colorectal cancer patients: independent of oncogenetic features. *J Cell Physiol.* (2019) 234:4768–77. 10.1002/jcp.27273 30370522

[60] Fridman WH Pagès F Sautès-Fridman C Galon J. The immune contexture in human tumours: impact on clinical outcome. *Nat Rev Cancer.* (2012) 12:298–306. 10.1038/nrc3245 22419253

[61] Galon J Costes A Sanchez-Cabo F Kirilovsky A Mlecnik B Lagorce-Pagès C Type, density, and location of immune cells within human colorectal tumors predict clinical outcome. *Science.* (2006) 313:1960–4. 10.1126/science.1129139 17008531

[62] Bruni D Angell HK Galon J. The immune contexture and immunoscore in cancer prognosis and therapeutic efficacy. *Nat Rev Cancer.* (2020) 20:662–80. 10.1038/s41568-020-0285-7 32753728

[63] Alspach E Lussier DM Schreiber RD. Interferon Γ and its important roles in promoting and inhibiting spontaneous and therapeutic cancer immunity. *Cold Spring Harb Perspect Biol.* (2019) 11:a028480. 10.1101/cshperspect.a028480 29661791 PMC6396335

[64] Liu Y Zhou N Zhou L Wang J Zhou Y Zhang T Il-2 regulates tumor-reactive Cd8(+) T cell exhaustion by activating the aryl hydrocarbon receptor. *Nat Immunol.* (2021) 22:358–69. 10.1038/s41590-020-00850-9 33432230

[65] Yu P Fu YX. Tumor-infiltrating T lymphocytes: friends or foes? *Lab Invest.* (2006) 86:231–45. 10.1038/labinvest.3700389 16446705

[66] Thommen DS Schumacher TN. T cell dysfunction in cancer. *Cancer Cell.* (2018) 33:547–62. 10.1016/j.ccell.2018.03.012 29634943 PMC7116508

[67] Zarour HM. Reversing T-cell dysfunction and exhaustion in cancer. *Clin Cancer Res.* (2016) 22:1856–64. 10.1158/1078-0432.Ccr-15-1849 27084739 PMC4872712

[68] Hua D Sun J Mao Y Chen LJ Wu YY Zhang XG. B7-H1 expression is associated with expansion of regulatory T cells in colorectal carcinoma. *World J Gastroenterol.* (2012) 18:971–8. 10.3748/wjg.v18.i9.971 22408358 PMC3297058

[69] Lin Y-C Mahalingam J Chiang J-M Su P-J Chu Y-Y Lai H-Y Activated but not resting regulatory T cells accumulated in tumor microenvironment and correlated with tumor progression in patients with colorectal cancer. *Int J Cancer.* (2013) 132:1341–50. 10.1002/ijc.27784 22907255

[70] Scurr M Ladell K Besneux M Christian A Hockey T Smart K Highly prevalent colorectal cancer-infiltrating Lap+ Foxp3- T cells exhibit more potent immunosuppressive activity than Foxp3+ regulatory T cells. *Mucosal Immunol.* (2014) 7:428–39. 10.1038/mi.2013.62 24064667 PMC3931584

[71] Camisaschi C Casati C Rini F Perego M De Filippo A Triebel F Lag-3 expression defines a subset of CD4(+)CD25(high)Foxp3(+) regulatory T cells that are expanded at tumor sites. *J Immunol.* (2010) 184:6545. 10.4049/jimmunol.0903879 20421648

[72] Blackburn SD Shin H Haining WN Zou T Workman CJ Polley A Coregulation of Cd8+ T cell exhaustion by multiple inhibitory receptors during chronic viral infection. *Nat Immunol.* (2009) 10:29–37. 10.1038/ni.1679 19043418 PMC2605166

[73] Xu B Yuan L Gao Q Yuan P Zhao P Yuan H Circulating and tumor-infiltrating tim-3 in patients with colorectal cancer. *Oncotarget.* (2015) 6:20592. 26008981 10.18632/oncotarget.4112 PMC4653028

[74] Arai Y Saito H Ikeguchi M. Upregulation of Tim-3 and Pd-1 on Cd4+ and Cd8+ T cells associated with dysfunction of cell-mediated immunity after colorectal cancer operation. *Yonago Acta Med.* (2012) 55:1. 24031134 PMC3727487

[75] Naidoo J Page DB Wolchok JD. Immune checkpoint blockade. *Hematol Oncol Clin North Am.* (2014) 28:585–600. 10.1016/j.hoc.2014.02.002 24880949

[76] Dyck L Mills KHG. Immune checkpoints and their inhibition in cancer and infectious diseases. *Eur J Immunol.* (2017) 47:765–79. 10.1002/eji.201646875 28393361

[77] Marcucci F Rumio C Corti A. Tumor cell-associated immune checkpoint molecules - drivers of malignancy and stemness. *Biochim Biophys Acta Rev Cancer.* (2017) 1868:571–83. 10.1016/j.bbcan.2017.10.006 29056539

[78] Pardoll DM. The blockade of immune checkpoints in cancer immunotherapy. *Nat Rev Cancer.* (2012) 12:252–64. 10.1038/nrc3239 22437870 PMC4856023

[79] Marisa L Svrcek M Collura A Becht E Cervera P Wanherdrick K The balance between cytotoxic T-cell lymphocytes and immune checkpoint expression in the prognosis of colon tumors. *J Natl Cancer Inst.* (2018) 110:68–77. 10.1093/jnci/djx136 28922790

[80] Tan E Sahin IH. Defining the current role of immune checkpoint inhibitors in the treatment of mismatch repair-deficient/microsatellite stability-high colorectal cancer and shedding light on future approaches. *Exp Rev Gastroenterol Hepatol.* (2021) 15:735–42. 10.1080/17474124.2021.1886077 33539189

[81] Le Flahec G Badic B Guibourg B Doucet L Bail JP Marcorelles P Mismatch repair-deficient colorectal cancer: a model of immunogenic and immune cell-rich tumor despite nonsignificant programmed cell death ligand-1 expression in tumor cells. *Hum Pathol.* (2018) 72:135–43. 10.1016/j.humpath.2017.09.019 29208565

[82] Michael-Robinson JM Biemer-Hüttmann A Purdie DM Walsh MD Simms LA Biden KG Tumour infiltrating lymphocytes and apoptosis are independent features in colorectal cancer stratified according to microsatellite instability status. *Gut.* (2001) 48:360–6. 10.1136/gut.48.3.360 11171826 PMC1760146

[83] Phillips SM Banerjea A Feakins R Li SR Bustin SA Dorudi S. Tumour-infiltrating lymphocytes in colorectal cancer with microsatellite instability are activated and cytotoxic. *Br J Surg.* (2004) 91:469–75. 10.1002/bjs.4472 15048750

[84] Llosa NJ Cruise M Tam A Wicks EC Hechenbleikner EM Taube JM The vigorous immune microenvironment of microsatellite instable colon cancer is balanced by multiple counter-inhibitory checkpoints. *Cancer Discov.* (2015) 5:43–51. 10.1158/2159-8290.Cd-14-0863 25358689 PMC4293246

[85] Dolcetti R Viel A Doglioni C Russo A Guidoboni M Capozzi E High prevalence of activated intraepithelial cytotoxic T lymphocytes and increased neoplastic cell apoptosis in colorectal carcinomas with microsatellite instability. *Am J Pathol.* (1999) 154:1805–13. 10.1016/s0002-9440(10)65436-3 10362805 PMC1866613

[86] Le DT Uram JN Wang H Bartlett BR Kemberling H Eyring AD PD-1 blockade in tumors with mismatch-repair deficiency. *New Engl J Med.* (2015) 372:2509–20. 10.1056/NEJMoa1500596 26028255 PMC4481136

[87] Jiang X Wang J Deng X Xiong F Ge J Xiang B Role of the tumor microenvironment in PD-L1/PD-1-mediated tumor immune escape. *Mol Cancer.* (2019) 18:10. 10.1186/s12943-018-0928-4 30646912 PMC6332843

[88] Dong Y Sun Q Zhang X. PD-1 and its ligands are important immune checkpoints in cancer. *Oncotarget.* (2017) 8:2171–86. 10.18632/oncotarget.13895 27974689 PMC5356790

[89] Taube JM Klein A Brahmer JR Xu H Pan X Kim JH Association of PD-1, PD-1 ligands, and other features of the tumor immune microenvironment with response to anti-PD-1 therapy. *Clin Cancer Res.* (2014) 20:5064–74. 10.1158/1078-0432.Ccr-13-3271 24714771 PMC4185001

[90] Gao L Guo Q Li X Yang X Ni H Wang T Mir-873/PD-L1 axis regulates the stemness of breast cancer cells. *EBioMedicine.* (2019) 41:395–407. 10.1016/j.ebiom.2019.02.034 30803931 PMC6444076

[91] Massari F Santoni M Ciccarese C Santini D Alfieri S Martignoni G PD-1 blockade therapy in renal cell carcinoma: current studies and future promises. *Cancer Treat Rev.* (2015) 41:114–21. 10.1016/j.ctrv.2014.12.013 25586601

[92] Yaghoubi N Soltani A Ghazvini K Hassanian SM Hashemy SI. PD-1/ PD-L1 blockade as a novel treatment for colorectal cancer. *Biomed Pharmacother.* (2019) 110:312–8. 10.1016/j.biopha.2018.11.105 30522017

[93] Li Y Liang L Dai W Cai G Xu Y Li X Prognostic impact of programed cell death-1 (PD-1) and PD-ligand 1 (PD-L1) expression in cancer cells and tumor infiltrating lymphocytes in colorectal cancer. *Mol Cancer.* (2016) 15:55. 10.1186/s12943-016-0539-x 27552968 PMC4995750

[94] Schalper KA Velcheti V Carvajal D Wimberly H Brown J Pusztai L In situ tumor PD-L1 mrna expression is associated with increased tils and better outcome in breast carcinomas. *Clin Cancer Res.* (2014) 20:2773–82. 10.1158/1078-0432.Ccr-13-2702 24647569

[95] Sabatier R Finetti P Mamessier E Adelaide J Chaffanet M Ali HR Prognostic and predictive value of PDL1 expression in breast cancer. *Oncotarget.* (2015) 6:5449–64. 10.18632/oncotarget.3216 25669979 PMC4467160

[96] Lipson EJ Sharfman WH Drake CG Wollner I Taube JM Anders RA Durable cancer regression off-treatment and effective reinduction therapy with an anti-PD-1 antibody. *Clin Cancer Res.* (2013) 19:462–8. 10.1158/1078-0432.CCR-12-2625 23169436 PMC3548952

[97] Chin Y Janseens J Vandepitte J Vandenbrande J Opdebeek L Raus J. Phenotypic analysis of tumor-infiltrating lymphocytes from human breast cancer. *Anticancer Res.* (1992) 12:1463–6. 1332579

[98] Nelson BH. CD20+ B cells: the other tumor-infiltrating lymphocytes. *J Immunol.* (2010) 185:4977–82. 10.4049/jimmunol.1001323 20962266

[99] Talaat IM Elemam NM Saber-Ayad M. Complement system: an immunotherapy target in colorectal cancer. *Front Immunol.* (2022) 13:810993. 10.3389/fimmu.2022.810993 35173724 PMC8841337

[100] Kinker GS Vitiello GAF Ferreira WAS Chaves AS Cordeiro de Lima VC Medina TDS. B cell orchestration of anti-tumor immune responses: a matter of cell localization and communication. *Front Cell Dev Biol.* (2021) 9:678127. 10.3389/fcell.2021.678127 34164398 PMC8215448

[101] Linnebacher M. Tumor-infiltrating B cells come into vogue. *World J Gastroenterol.* (2013) 19:8–11. 10.3748/wjg.v19.i1.8 23326156 PMC3542760

[102] Yasuda M Mizukami M Hanagiri T Shigematsu Y Fukuyama T Nagata Y Antigens recognized by igg derived from tumor-infiltrating B lymphocytes in human lung cancer. *Anticancer Res.* (2006) 26:3607–11. 17094490

[103] Meshcheryakova A Tamandl D Bajna E Stift J Mittlboeck M Svoboda M B cells and ectopic follicular structures: novel players in anti-tumor programming with prognostic power for patients with metastatic colorectal cancer. *PLoS One.* (2014) 9:e99008. 10.1371/journal.pone.0099008 24905750 PMC4048213

[104] Maletzki C Jahnke A Ostwald C Klar E Prall F Linnebacher M. Ex-vivo clonally expanded B lymphocytes infiltrating colorectal carcinoma are of mature immunophenotype and produce functional igg. *PLoS One.* (2012) 7:e32639. 10.1371/journal.pone.0032639 22393427 PMC3290587

[105] Ogino S Nosho K Irahara N Meyerhardt JA Baba Y Shima K Lymphocytic reaction to colorectal cancer is associated with longer survival, independent of lymph node count, microsatellite instability, and CpG island methylator phenotype. *Clin Cancer Res.* (2009) 15:6412–20. 10.1158/1078-0432.CCR-09-1438 19825961 PMC2771425

[106] Deschoolmeester V Baay M Van Marck E Weyler J Vermeulen P Lardon F Tumor infiltrating lymphocytes: an intriguing player in the survival of colorectal cancer patients. *BMC Immunol.* (2010) 11:19. 10.1186/1471-2172-11-19 20385003 PMC2864219

[107] Freeman GJ Long AJ Iwai Y Bourque K Chernova T Nishimura H Engagement of the PD-1 immunoinhibitory receptor by a novel B7 family member leads to negative regulation of lymphocyte activation. *J Exp Med.* (2000) 192:1027–34. 10.1084/jem.192.7.1027 11015443 PMC2193311

[108] Vasilevko V Ghochikyan A Holterman MJ Agadjanyan MG. CD80 (B7-1) and CD86 (B7-2) are functionally equivalent in the initiation and maintenance of CD4+ T-cell proliferation after activation with suboptimal doses of PHA. *DNA Cell Biol.* (2002) 21:137–49. 10.1089/10445490252925404 12015893

[109] Wang F Zhu W Liu T Sun Z Ju S Ju S The expression analysis of icos-L on activated T cells and immature dendritic cells as well as malignant B cells and Grave’s-disease-derived thyroid tissues by two novel mAbs against human ICOS-L. *Tissue Antigens.* (2007) 69:62–72. 10.1111/j.1399-0039.2006.00706.x 17212709

[110] Helmink BA Reddy SM Gao J Zhang S Basar R Thakur R B cells and tertiary lymphoid structures promote immunotherapy response. *Nature.* (2020) 577:549–55. 10.1038/s41586-019-1922-8 31942075 PMC8762581

[111] Carrega P Bonaccorsi I Di Carlo E Morandi B Paul P Rizzello V Cd56(Bright) perforin(low)noncytotoxic human Nk cells are abundant in both healthy and neoplastic solid tissues and recirculate to secondary lymphoid organs via afferent lymph. *J Immunol.* (2014) 192:3805. 10.4049/jimmunol.1301889 24646734

[112] Cooper MA Fehniger TA Turner SC Chen KS Ghaheri BA Ghayur T Human natural killer cells: a unique innate immunoregulatory role for the CD56(bright) subset. *Blood.* (2001) 97:3146–51. 10.1182/blood.v97.10.3146 11342442

[113] Maghazachi AA. Compartmentalization of human natural killer cells. *Mol Immunol.* (2005) 42:523–9. 10.1016/j.molimm.2004.07.036 15607808

[114] Huntington ND Cursons J Rautela J. The cancer-natural killer cell immunity cycle. *Nat Rev Cancer.* (2020) 20:437–54. 10.1038/s41568-020-0272-z 32581320

[115] O’Sullivan T Saddawi-Konefka R Vermi W Koebel CM Arthur C White JM Cancer immunoediting by the innate immune system in the absence of adaptive immunity. *J Exp Med.* (2012) 209:1869–82. 10.1084/jem.20112738 22927549 PMC3457735

[116] O’Brien KL Finlay DK. Immunometabolism and natural killer cell responses. *Nat Rev Immunol.* (2019) 19:282–90. 10.1038/s41577-019-0139-2 30808985

[117] Habif G Crinier A André P Vivier E Narni-Mancinelli E. Targeting natural killer cells in solid tumors. *Cell Mol Immunol.* (2019) 16:415–22. 10.1038/s41423-019-0224-2 30911118 PMC6474204

[118] Konjević GM Vuletić AM Mirjačić Martinović KM Larsen AK Jurišić VB. The role of cytokines in the regulation of Nk cells in the tumor environment. *Cytokine.* (2019) 117:30–40. 10.1016/j.cyto.2019.02.001 30784898

[119] Hornyák L Dobos N Koncz G Karányi Z Páll D Szabó Z The role of indoleamine-2,3-dioxygenase in cancer development, diagnostics, and therapy. *Front Immunol.* (2018) 9:151. 10.3389/fimmu.2018.00151 29445380 PMC5797779

[120] Fruci D Monaco EL Cifaldi L Locatelli F Tremante E Benevolo M T and Nk cells: two sides of tumor immunoevasion. *J Transl Med.* (2013) 11:30. 10.1186/1479-5876-11-30 23379575 PMC3621684

[121] Paul S Kulkarni N Shilpi, Lal G. Intratumoral natural killer cells show reduced effector and cytolytic properties and control the differentiation of effector Th1 cells. *Oncoimmunology.* (2016) 5:e1235106. 10.1080/2162402X.2016.1235106 28151533 PMC5214058

[122] Viel S Marçais A Guimaraes FS-F Loftus R Rabilloud J Grau M Tgf-B inhibits the activation and functions of Nk cells by repressing the Mtor pathway. *Sci Signal.* (2016) 9:ra19. 10.1126/scisignal.aad1884 26884601

[123] Clift R Souratha J Garrovillo SA Zimmerman S Blouw B. Remodeling the tumor microenvironment sensitizes breast tumors to anti-programmed death-ligand 1 immunotherapy. *Cancer Res.* (2019) 79:4149–59. 10.1158/0008-5472.CAN-18-3060 31248966

[124] Andersson E Poschke I Villabona L Carlson JW Lundqvist A Kiessling R Non-classical Hla-class I expression in serous ovarian carcinoma: correlation with the Hla-Genotype, tumor infiltrating immune cells and prognosis. *Oncoimmunology.* (2015) 5:e1052213. 10.1080/2162402X.2015.1052213 26942060 PMC4760332

[125] Kamiya T Seow SV Wong D Robinson M Campana D. Blocking expression of inhibitory receptor Nkg2a overcomes tumor resistance to Nk cells. *J Clin Invest.* (2019) 129:2094–106. 10.1172/JCI123955 30860984 PMC6486333

[126] Braud VM Allan DS O’Callaghan CA Söderström K D’Andrea A Ogg GS Hla-E binds to natural killer cell receptors Cd94/Nkg2a, B and C. *Nature.* (1998) 391:795–9. 10.1038/35869 9486650

[127] Malmberg K-J Carlsten M Björklund A Sohlberg E Bryceson YT Ljunggren H-G. Natural killer cell-mediated immunosurveillance of human cancer. *Semin Immunol.* (2017) 31:20–9. 10.1016/j.smim.2017.08.002 28888619

[128] Concha-Benavente F Kansy B Moskovitz J Moy J Chandran U Ferris RL. Pd-L1 mediates dysfunction in activated Pd-1(+) Nk cells in head and neck cancer patients. *Cancer Immunol Res.* (2018) 6:1548–60. 10.1158/2326-6066.CIR-18-0062 30282672 PMC6512340

[129] Hsu J Hodgins JJ Marathe M Nicolai CJ Bourgeois-Daigneault M-C Trevino TN Contribution of Nk cells to immunotherapy mediated by Pd-1/Pd-L1 blockade. *J Clin Invest.* (2018) 128:4654–68. 10.1172/JCI99317 30198904 PMC6159991

[130] Choucair K Duff JR Cassidy CS Albrethsen MT Kelso JD Lenhard A Natural killer cells: a review of biology, therapeutic potential and challenges in treatment of solid tumors. *Future Oncol.* (2019) 15:3053–69. 10.2217/fon-2019-0116 31411057

[131] Pivarcsi A Müller A Hippe A Rieker J van Lierop A Steinhoff M Tumor immune escape by the loss of homeostatic chemokine expression. *Proc Natl Acad Sci U.S.A.* (2007) 104:19055–60. 10.1073/pnas.0705673104 18025475 PMC2141907

[132] Molon B Ugel S Del Pozzo F Soldani C Zilio S Avella D Chemokine nitration prevents intratumoral infiltration of antigen-specific T cells. *J Exp Med.* (2011) 208:1949–62. 10.1084/jem.20101956 21930770 PMC3182051

[133] Proost P Mortier A Loos T Vandercappellen J Gouwy M Ronsse I Proteolytic processing of Cxcl11 by Cd13/aminopeptidase N impairs Cxcr3 and Cxcr7 binding and signaling and reduces lymphocyte and endothelial cell migration. *Blood.* (2007) 110:37–44. 10.1182/blood-2006-10-049072 17363734

[134] Sandel MH Speetjens FM Menon AG Albertsson PA Basse PH Hokland M Natural killer cells infiltrating colorectal cancer and Mhc class I expression. *Mol Immunol.* (2005) 42:541–6. 10.1016/j.molimm.2004.07.039 15607811

[135] Menon AG Morreau H Tollenaar RAEM Alphenaar E Van Puijenbroek M Putter H Down-regulation of Hla-a expression correlates with a better prognosis in colorectal cancer patients. *Lab Invest.* (2002) 82:1725–33. 10.1097/01.lab.0000043124.75633.ed 12480922

[136] Atreya I Neurath MF. Immune cells in colorectal cancer: prognostic relevance and therapeutic strategies. *Expert Rev Anticancer Ther.* (2008) 8:561–72. 10.1586/14737140.8.4.561 18402523

[137] Jobin G Rodriguez-Suarez R Betito K. Association between natural killer cell activity and colorectal cancer in high-risk subjects undergoing colonoscopy. *Gastroenterology.* (2017) 153:980–7. 10.1053/j.gastro.2017.06.009 28625834

[138] Wang Y Sun J Gao W Song B Shao Q Zhao L Preoperative Tim−3 expression on peripheral Nk cells is correlated with pathologic Tnm staging in colorectal cancer. *Mol Med Rep.* (2017) 15:3810–8. 10.3892/mmr.2017.6482 28440449

[139] Krijgsman D de Vries NL Skovbo A Andersen MN Swets M Bastiaannet E Characterization of circulating T-, Nk-, and Nkt cell subsets in patients with colorectal cancer: the peripheral blood immune cell profile. *Cancer Immunol Immunother.* (2019) 68:1011–24. 10.1007/s00262-019-02343-7 31053876 PMC6529387

[140] Rocca YS Roberti MP Arriaga JM Amat M Bruno L Pampena MB Altered phenotype in peripheral blood and tumor-associated Nk cells from colorectal cancer patients. *Innate Immunity.* (2012) 19:76–85. 10.1177/1753425912453187 22781631

[141] Peng Y-P Zhu Y Zhang J-J Xu Z-K Qian Z-Y Dai C-C Comprehensive analysis of the percentage of surface receptors and cytotoxic granules positive natural killer cells in patients with pancreatic cancer, gastric cancer, and colorectal cancer. *J Transl Med.* (2013) 11:262. 10.1186/1479-5876-11-262 24138752 PMC3854023

[142] Doubrovina ES Doubrovin MM Vider E Sisson RB O’Reilly RJ Dupont B Evasion from Nk Cell immunity by Mhc class I chain-related molecules expressing colon adenocarcinoma. *J Immunol.* (2003) 171:6891–9. 10.4049/jimmunol.171.12.6891 14662896

[143] Gharagozloo M Kalantari H Rezaei A Maracy MR Salehi M Bahador A The decrease in Nkg2d+ natural killer cells in peripheral blood of patients with metastatic colorectal cancer. *Bratisl Lek Listy.* (2015) 116:296–301. 10.4149/bll_2015_056 25924638

[144] Arreygue-Garcia NA Daneri-Navarro A del Toro-Arreola A Cid-Arregui A Gonzalez-Ramella O Jave-Suarez LF Augmented serum level of major histocompatibility complex class I-related chain a (Mica) protein and reduced Nkg2d expression on Nk and T cells in patients with cervical cancer and precursor lesions. *BMC Cancer.* (2008) 8:16. 10.1186/1471-2407-8-16 18208618 PMC2270854

[145] Tsushima H Kawata S Tamura S Ito N Shirai Y Kiso S High levels of transforming growth factor beta 1 in patients with colorectal cancer: association with disease progression. *Gastroenterology.* (1996) 110:375–82. 10.1053/gast.1996.v110.pm8566583 8566583

[146] McGilvray RW Eagle RA Watson NFS Al-Attar A Ball G Jafferji I Nkg2d ligand expression in human colorectal cancer reveals associations with prognosis and evidence for immunoediting. *Clin Cancer Res.* (2009) 15:6993. 10.1158/1078-0432.CCR-09-0991 19861434 PMC2778653

[147] Cui F Qu D Sun R Tao H Si J Xu Y. The role of circulating Cd16+Cd56+ natural killer cells in the screening, diagnosis, and staging of colorectal cancer before initial treatment. *Dis Markers.* (2019) 2019:7152183. 10.1155/2019/7152183 31636738 PMC6766087

[148] Zhang C Liu Y. Targeting Nk cell checkpoint receptors or molecules for cancer immunotherapy. *Front Immunol.* (2020) 11:1295. 10.3389/fimmu.2020.01295 32714324 PMC7344328

[149] André P Denis C Soulas C Bourbon-Caillet C Lopez J Arnoux T Anti-Nkg2a Mab Is a checkpoint inhibitor that promotes anti-tumor immunity by unleashing both T and Nk cells. *Cell.* (2018) 175:1731–43.e13. 10.1016/j.cell.2018.10.014 30503213 PMC6292840

[150] Tallerico R Todaro M Di Franco S Maccalli C Garofalo C Sottile R Human Nk cells selective targeting of colon cancer-initiating cells: a role for natural cytotoxicity receptors and Mhc class I molecules. *J Immunol.* (2013) 190:2381–90. 10.4049/jimmunol.1201542 23345327

[151] Oh S Lee J-H Kwack K Choi S-W. Natural killer cell therapy: a new treatment paradigm for solid tumors. *Cancers (Basel).* (2019) 11:1534. 10.3390/cancers11101534 31614472 PMC6826624

[152] Abel AM Yang C Thakar MS Malarkannan S. Natural killer cells: development, maturation, and clinical utilization. *Front Immunol.* (2018) 9:1869. 10.3389/fimmu.2018.01869 30150991 PMC6099181

[153] Hu W Wang G Huang D Sui M Xu Y. Cancer immunotherapy based on natural killer cells: current progress and new opportunities. *Front Immunol.* (2019) 10:1205. 10.3389/fimmu.2019.01205 31214177 PMC6554437

[154] Ishikawa T Okayama T Sakamoto N Ideno M Oka K Enoki T Phase I clinical trial of adoptive transfer of expanded natural killer cells in combination with Igg1 antibody in patients with gastric or colorectal cancer. *Int J Cancer.* (2018) 142:2599–609. 10.1002/ijc.31285 29388200

[155] Veluchamy JP Spanholtz J Tordoir M Thijssen VL Heideman DAM Verheul HMW Combination of Nk cells and cetuximab to enhance anti-tumor responses in Ras mutant metastatic colorectal cancer. *PLoS One.* (2016) 11:e0157830. 10.1371/journal.pone.0157830 27314237 PMC4912059

[156] Larsen SK Gao Y Basse PH. Nk cells in the tumor microenvironment. *Crit Rev Oncog.* (2014) 19:91–105. 10.1615/critrevoncog.2014011142 24941376 PMC4062922

[157] Halama N Braun M Kahlert C Spille A Quack C Rahbari N Natural killer cells are scarce in colorectal carcinoma tissue despite high levels of chemokines and cytokines. *Clin Cancer Res.* (2011) 17:678–89. 10.1158/1078-0432.CCR-10-2173 21325295

[158] Khan M Arooj S Wang H. Nk cell-based immune checkpoint inhibition. *Front Immunol.* (2020) 11:167. 10.3389/fimmu.2020.00167 32117298 PMC7031489

[159] Zhang Q Bi J Zheng X Chen Y Wang H Wu W Blockade of the checkpoint receptor tigit prevents Nk cell exhaustion and elicits potent anti-tumor immunity. *Nat Immunol.* (2018) 19:723–32. 10.1038/s41590-018-0132-0 29915296

[160] Dixon KO Schorer M Nevin J Etminan Y Amoozgar Z Kondo T Functional anti-tigit antibodies regulate development of autoimmunity and antitumor immunity. *J Immunol.* (2018) 200:3000–7. 10.4049/jimmunol.1700407 29500245 PMC5893394

[161] Liu Y Cheng Y Xu Y Wang Z Du X Li C Increased expression of programmed cell death protein 1 on Nk cells inhibits Nk-cell-mediated anti-tumor function and indicates poor prognosis in digestive cancers. *Oncogene.* (2017) 36:6143–53. 10.1038/onc.2017.209 28692048 PMC5671935

[162] Crocker PR Paulson JC Varki A. Siglecs and their roles in the immune system. *Nat Rev Immunol.* (2007) 7:255–66. 10.1038/nri2056 17380156

[163] Jandus C Boligan KF Chijioke O Liu H Dahlhaus M Démoulins T Interactions between Siglec-7/9 receptors and ligands influence Nk cell-dependent tumor immunosurveillance. *J Clin Invest.* (2014) 124:1810–20. 10.1172/jci65899 24569453 PMC3973073

[164] Bénac O Gaudin M Ors M Roy AL Blanc HR Soulas C Abstract 2713: preclinical development of first-in-class antibodies targeting Siglec-9 immune checkpoint for cancer immunotherapy. *Cancer Res.* (2018) 78(13 Suppl.):2713. 10.1158/1538-7445.AM2018-2713

[165] Stanczak MA Siddiqui SS Trefny MP Thommen DS Boligan KF von Gunten S Self-associated molecular patterns mediate cancer immune evasion by engaging Siglecs on T cells. *J Clin Invest.* (2018) 128:4912–23. 10.1172/jci120612 30130255 PMC6205408

[166] Zhong X Chen B Yang Z. The role of tumor-associated macrophages in colorectal carcinoma progression. *Cell Physiol Biochem.* (2018) 45:356–65. 10.1159/000486816 29402795

[167] Jayasingam SD Citartan M Thang TH Mat Zin AA Ang KC Ch’ng ES. Evaluating the polarization of tumor-associated macrophages into M1 and M2 phenotypes in human cancer tissue: technicalities and challenges in routine clinical practice. *Front Oncol.* (2020) 9:1512. 10.3389/fonc.2019.01512 32039007 PMC6992653

[168] Yahaya MAF Lila MAM Ismail S Zainol M Afizan N. Tumour-associated macrophages (Tams) in colon cancer and how to reeducate them. *J Immunol Res.* (2019) 2019:2368249. 10.1155/2019/2368249 30931335 PMC6410439

[169] Kessenbrock K Plaks V Werb Z. Matrix metalloproteinases: regulators of the tumor microenvironment. *Cell.* (2010) 141:52–67. 10.1016/j.cell.2010.03.015 20371345 PMC2862057

[170] Prenen H Mazzone M. Tumor-associated macrophages: a short compendium. *Cell Mol Life Sci.* (2019) 76:1447–58. 10.1007/s00018-018-2997-3 30747250 PMC11105658

[171] Pinto ML Rios E Silva AC Neves SC Caires HR Pinto AT Decellularized human colorectal cancer matrices polarize macrophages towards an anti-inflammatory phenotype promoting cancer cell invasion via Ccl18. *Biomaterials.* (2017) 124:211–24. 10.1016/j.biomaterials.2017.02.004 28209528

[172] Marech I Ammendola M Sacco R Sammarco G Zuccalà V Zizzo N Tumour-associated macrophages correlate with microvascular bed extension in colorectal cancer patients. *J Cell Mol Med.* (2016) 20:1373–80. 10.1111/jcmm.12826 27105577 PMC4929299

[173] Mantovani A Marchesi F Malesci A Laghi L Allavena P. Tumour-associated macrophages as treatment targets in oncology. *Nat Rev Clin Oncol.* (2017) 14:399–416. 10.1038/nrclinonc.2016.217 28117416 PMC5480600

[174] DeNardo DG Brennan DJ Rexhepaj E Ruffell B Shiao SL Madden SF Leukocyte complexity predicts breast cancer survival and functionally regulates response to chemotherapy. *Cancer Discov.* (2011) 1:54–67. 10.1158/2159-8274.Cd-10-0028 22039576 PMC3203524

[175] Pyonteck SM Akkari L Schuhmacher AJ Bowman RL Sevenich L Quail DF Csf-1r inhibition alters macrophage polarization and blocks glioma progression. *Nat Med.* (2013) 19:1264–72. 10.1038/nm.3337 24056773 PMC3840724

[176] Ries CH Cannarile MA Hoves S Benz J Wartha K Runza V Targeting tumor-associated macrophages with anti-Csf-1r antibody reveals a strategy for cancer therapy. *Cancer Cell.* (2014) 25:846–59. 10.1016/j.ccr.2014.05.016 24898549

[177] Malfitano AM Pisanti S Napolitano F Di Somma S Martinelli R Portella G. Tumor-associated macrophage status in cancer treatment. *Cancers (Basel).* (2020) 12:1987. 10.3390/cancers12071987 32708142 PMC7409350

[178] Zhao C Pang X Yang Z Wang S Deng H Chen X. Nanomaterials targeting tumor associated macrophages for cancer immunotherapy. *J Control Release.* (2022) 341:272–84. 10.1016/j.jconrel.2021.11.028 34813877

[179] Fiegle E Doleschel D Koletnik S Rix A Weiskirchen R Borkham-Kamphorst E Dual Ctla-4 and Pd-L1 blockade inhibits tumor growth and liver metastasis in a highly aggressive orthotopic mouse model of colon cancer. *Neoplasia.* (2019) 21:932–44. 10.1016/j.neo.2019.07.006 31412307 PMC6700499

[180] Kinouchi M Miura K Mizoi T Ishida K Fujibuchi W Sasaki H Infiltration of CD40-positive tumor-associated macrophages indicates a favorable prognosis in colorectal cancer patients. *Hepato Gastroenterol.* (2013) 60:83–8. 10.5754/hge12372 22687258

[181] Dallman C Johnson PW Packham G. Differential regulation of cell survival by CD40. *Apoptosis.* (2003) 8:45–53. 10.1023/a:1021696902187 12510151

[182] Georgopoulos NT Merrick A Scott N Selby PJ Melcher A Trejdosiewicz LK. CD40-mediated death and cytokine secretion in colorectal cancer: a potential target for inflammatory tumour cell killing. *Int J Cancer.* (2007) 121:1373–81. 10.1002/ijc.22846 17534894

[183] Coffelt SB Wellenstein MD de Visser KE. Neutrophils in cancer: neutral no more. *Nat Rev Cancer.* (2016) 16:431–46. 10.1038/nrc.2016.52 27282249

[184] Fridlender ZG Sun J Kim S Kapoor V Cheng G Ling L Polarization of tumor-associated neutrophil phenotype by Tgf-beta: “N1” versus “N2” Tan. *Cancer Cell.* (2009) 16:183–94. 10.1016/j.ccr.2009.06.017 19732719 PMC2754404

[185] Ohms M Möller S Laskay T. An attempt to polarize human neutrophils toward N1 and N2 phenotypes in vitro. *Front Immunol.* (2020) 11:532. 10.3389/fimmu.2020.00532 32411122 PMC7198726

[186] Shaul ME Fridlender ZG. Tumour-associated neutrophils in patients with cancer. *Nat Rev Clin Oncol.* (2019) 16:601–20. 10.1038/s41571-019-0222-4 31160735

[187] Jaillon S Ponzetta A Di Mitri D Santoni A Bonecchi R Mantovani A. Neutrophil diversity and plasticity in tumour progression and therapy. *Nat Rev Cancer.* (2020) 20:485–503. 10.1038/s41568-020-0281-y 32694624

[188] Finisguerra V Di Conza G Di Matteo M Serneels J Costa S Thompson AA Met is required for the recruitment of anti-tumoural neutrophils. *Nature.* (2015) 522:349–53. 10.1038/nature14407 25985180 PMC4594765

[189] Jablonska J Leschner S Westphal K Lienenklaus S Weiss S. Neutrophils responsive to endogenous Ifn-beta regulate tumor angiogenesis and growth in a mouse tumor model. *J Clin Invest.* (2010) 120:1151–64. 10.1172/jci37223 20237412 PMC2846036

[190] Bonecchi R Mantovani A Jaillon S. Chemokines as regulators of neutrophils: focus on tumors. *Therap Target Immunother.* (2022) 14:680. 10.3390/cancers14030680 PMC8833344 35158948

[191] Grzywa TM Sosnowska A Matryba P Rydzynska Z Jasinski M Nowis D Myeloid cell-derived arginase in cancer immune response. *Front Immunol.* (2020) 11:938. 10.3389/fimmu.2020.00938 32499785 PMC7242730

[192] Andzinski L Kasnitz N Stahnke S Wu CF Gereke M von Köckritz-Blickwede M Type I Ifns induce anti-tumor polarization of tumor associated neutrophils in mice and human. *Int J Cancer.* (2016) 138:1982–93. 10.1002/ijc.29945 26619320

[193] Raccosta L Fontana R Maggioni D Lanterna C Villablanca EJ Paniccia A The oxysterol-Cxcr2 axis plays a key role in the recruitment of tumor-promoting neutrophils. *J Exp Med.* (2013) 210:1711–28. 10.1084/jem.20130440 23897983 PMC3754872

[194] Reis ES Mastellos DC Ricklin D Mantovani A Lambris JD. Complement in cancer: untangling an intricate relationship. *Nat Rev Immunol.* (2018) 18:5–18. 10.1038/nri.2017.97 28920587 PMC5816344

[195] Mollica Poeta V Massara M Capucetti A Bonecchi R. Chemokines and chemokine receptors: new targets for cancer immunotherapy. *Front Immunol.* (2019) 10:379. 10.3389/fimmu.2019.00379 30894861 PMC6414456

[196] Tohme S Yazdani HO Al-Khafaji AB Chidi AP Loughran P Mowen K Neutrophil extracellular traps promote the development and progression of liver metastases after surgical stress. *Cancer Res.* (2016) 76:1367–80. 10.1158/0008-5472.Can-15-1591 26759232 PMC4794393

[197] van der Windt DJ Sud V Zhang H Varley PR Goswami J Yazdani HO Neutrophil extracellular traps promote inflammation and development of hepatocellular carcinoma in nonalcoholic steatohepatitis. *Hepatology.* (2018) 68:1347–60. 10.1002/hep.29914 29631332 PMC6173613

[198] Berger-Achituv S Brinkmann V Abed UA Kühn LI Ben-Ezra J Elhasid R A proposed role for neutrophil extracellular traps in cancer immunoediting. *Front Immunol.* (2013) 4:48. 10.3389/fimmu.2013.00048 23508552 PMC3589747

[199] Galdiero MR Bianchi P Grizzi F Di Caro G Basso G Ponzetta A Occurrence and significance of tumor-associated neutrophils in patients with colorectal cancer. *Int J Cancer.* (2016) 139:446–56. 10.1002/ijc.30076 26939802

[200] Posabella A Köhn P Lalos A Wilhelm A Mechera R Soysal S High density of Cd66b in primary high-grade ovarian cancer independently predicts response to chemotherapy. *J Cancer Res Clin Oncol.* (2020) 146:127–36. 10.1007/s00432-019-03108-6 31853662 PMC11804757

[201] Germann M Zangger N Sauvain M-O Sempoux C Bowler AD Wirapati P Neutrophils suppress tumor-infiltrating T cells in colon cancer via matrix metalloproteinase-mediated activation of Tgfβ. *EMBO Mol Med.* (2020) 12:e10681. 10.15252/emmm.201910681 31793740 PMC6949488

[202] Jakubowska K Koda M Kisielewski W Kañczuga-Koda L Grudziñska M Famulski W. Pre- and postoperative neutrophil and lymphocyte count and neutrophil-to-lymphocyte ratio in patients with colorectal cancer. *Mol Clin Oncol.* (2020) 13:56. 10.3892/mco.2020.2126 32905328 PMC7468214

[203] Scapini P Morini M Tecchio C Minghelli S Di Carlo E Tanghetti E Cxcl1/macrophage inflammatory protein-2-induced angiogenesis in vivo is mediated by neutrophil-derived vascular endothelial growth factor-A. *J Immunol.* (2004) 172:5034–40. 10.4049/jimmunol.172.8.5034 15067085

[204] Li J Byrne KT Yan F Yamazoe T Chen Z Baslan T Tumor cell-intrinsic factors underlie heterogeneity of immune cell infiltration and response to immunotherapy. *Immunity.* (2018) 49:178–93.e7. 10.1016/j.immuni.2018.06.006 29958801 PMC6707727

[205] Liao W Overman MJ Boutin AT Shang X Zhao D Dey P Kras-Irf2 axis drives immune suppression and immune therapy resistance in colorectal cancer. *Cancer Cell.* (2019) 35:559–72.e7. 10.1016/j.ccell.2019.02.008 30905761 PMC6467776

[206] Hanahan D Weinberg RA. Hallmarks of cancer: the next generation. *Cell.* (2011) 144:646–74. 10.1016/j.cell.2011.02.013 21376230

[207] Faget J Peters S Quantin X Meylan E Bonnefoy N. Neutrophils in the era of immune checkpoint blockade. *J Immunother Cancer.* (2021) 9:e002242. 10.1136/jitc-2020-002242 34301813 PMC8728357

[208] Zhang H Wang Y Onuma A He J Wang H Xia Y Neutrophils extracellular traps inhibition improves Pd-1 blockade immunotherapy in colorectal cancer. *Cancers (Basel).* (2021) 13:5333. 10.3390/cancers13215333 34771497 PMC8582562

